# A new melilite-type rare-earth borate CdTbGaB_2_O_7_ and multicolor tunable emission in the CdTb_1−*x*_Sm_*x*_GaB_2_O_7_ (0 ≤ *x* ≤ 0.2) phosphors†

**DOI:** 10.1039/d3ra03002d

**Published:** 2023-06-01

**Authors:** Xuean Chen, Jinyuan Zhang, Weiqiang Xiao, Xiaoyan Song

**Affiliations:** a Faculty of Materials and Manufacturing, Key Laboratory of Advanced Functional Materials, Ministry of Education of China, Beijing University of Technology 100124 Beijing China xueanchen@bjut.edu.cn; b Beijing Key Laboratory of Microstructure and Property of Solids, Beijing University of Technology 100124 Beijing China

## Abstract

A new mixed metal borate, CdTbGaB_2_O_7_, was successfully synthesized using the high-temperature solution method and its crystal structure was determined by single-crystal X-ray diffraction with the following unit-cell data: *P*4̄2_1_*m*, *a* = *b* = 7.3487(1) Å, *c* = 4.7247(1) Å, *V* = 255.150(9) Å^3^, and *Z* = 2. It belongs to a new member of the melilite family, which features a 3D framework consisting of alternately stacked [Ga(B_2_O_7_)]_*n*_^5*n*−^ tetrahedral layers and (Cd^2+^/Tb^3+^) cationic layers that are interconnected *via* B(Ga)–O–(Cd/Tb) bridges. In addition, the solid solutions of CdTb_1−*x*_Sm_*x*_GaB_2_O_7_ (0 ≤ *x* ≤ 0.2) were prepared *via* the solid-state reaction method. The combined techniques of XRD, SEM, IR/Raman, XPS and PLE/PL were employed to characterize the products. It was found that the CdTb_1−*x*_Sm_*x*_GaB_2_O_7_ phosphors simultaneously showed green emission of Tb^3+^ at 545 nm and orange emission of Sm^3+^ at 603 nm under excitation at 370 nm. The emission color can be adjusted from green to orange-red by varying the Sm^3+^ doped content *via* an energy transfer mechanism. For CdTb_0.995_Sm_0.005_GaB_2_O_7_, a QY of 13.22% was obtained, and its emission intensity at 423 K was 94% of that at 303 K. These results show that the prepared materials can act as potential color-tunable phosphors for UV w-LEDs.

## Abstract 2

A new borate CdTbGaB_2_O_7_ was synthesized and characterized and luminescence properties of CdTb_1−*x*_Sm_*x*_GaB_2_O_7_ (0 ≤ *x* ≤ 0.2) were investigated.

## Graphical abstract

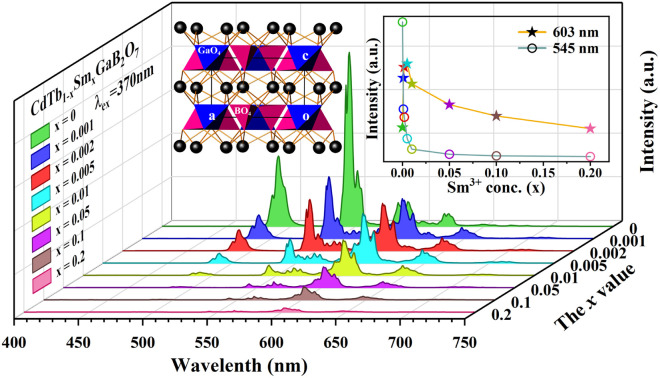

## 1. Introduction

Melilites include a large class of natural and synthetic compounds, whose general formula is A_2_XZ_2_O_7_, where A cations (A = Na, Ca, Sr, Ba, Cd, Pb, Y, Ln) are sandwiched between XZ_2_O_7_ tetrahedral layers (X = Be, Mg, Co, Fe, Mn, Cu, Zn, Cd, Al, Ga; Z = Be, B, Al, Ga, Si, Ge).^1^ The melilite-like compounds have been extensively investigated in the past due to their crystal chemical properties and potential applications. For example, crystallographic studies indicated that the stability of the melilite structure depends strongly on the degree of the size misfit between the tetrahedral layers and the interlayer cations.^2^ As far as their application prospects are concerned, some Si (or Ge)-based melilites may act as potential microwave dielectric materials.^3^ Doped with Nd^3+^, crystals of the melilite family, *e.g.* Ca_2_Ga_2_SiO_7_, Ba_2_MgGe_2_O_7_ and Ba_2_ZnGe_2_O_7_, were successfully used in high-power lasers, both with Xe-flash lamp pumping and with laser-diode pumping.^4–6^

The mineral okayamalite, Ca_2_SiB_2_O_7_, was first discovered by Giuli *et al.* in 2000, which is the only melilite borate known at early times.^7^ Subsequently, Barbier *et al.* conducted a systematic survey of several MO–Bi_2_O_3_ (and Ga_2_O_3_)–B_2_O_3_ systems and found three new diborate members of this family, including Bi_2_ZnB_2_O_7_, CaBiGaB_2_O_7_, and CdBiGaB_2_O_7_ (only unit-cell data were provided for the last borate).^8^ Among them, two Ga-containing compounds crystallize with the normal tetragonal melilite structure, whereas the Zn-containing phase adopts a unique orthorhombic superstructure of melilite. The attempted solid-state syntheses of the other melilites, such as Bi_2_MB_2_O_7_ (M = Be, Mg, Co), MBiGaB_2_O_7_ (M = Mg, Sr, Ba), CaBiMB_2_O_7_ (M = B, Al, In), and MBiZnB_2_O_7_ (M = Y, Nd, Yb), were unsuccessful. The preliminary measurements of second-harmonic generation (SHG) efficiencies (*d*_eff_) on powder samples yielded values of 4.0 (Bi_2_ZnB_2_O_7_) and 1.6 (CaBiGaB_2_O_7_) relative to a KH_2_PO_4_ (KDP) standard. The larger efficiency of Bi_2_ZnB_2_O_7_ was ascribed to the presence of planar BO_3_ groups and a higher concentration of the heavy and polarizable Bi^3+^ cations in its crystal structure. Soon after, relatively large single crystals of Bi_2_ZnB_2_O_7_ were successfully grown from a high-temperature melt by the top-seeded method, which reveals that this borate is a promising candidate for nonlinear optical (NLO) materials.^9^ This Zn-containing melilite diborate is still of current interest due to its potential value as a host to prepare different kinds of luminescent materials. For instance, Tb^3+^, Sm^3+^, Eu^3+^ and Dy^3+^-doped Bi_2_ZnB_2_O_7_ phosphors were synthesized and their luminescent properties were investigated.^10–12^ When active laser medium (such as Er^3+^, Nd^3+^ or Pr^3+^ ions) are doped into this crystal structure, the obtained crystals may show both luminescence and NLO properties, which makes them very attractive for the new generation of laser frequency converters, the representative crystals including Bi_2_ZnOB_2_O_6_:Yb^3+^/Er^3+^, Bi_2_ZnOB_2_O_6_:Nd^3+^ and Bi_2_ZnOB_2_O_6_:Pr^3+^.^13–15^ In addition, not long ago, two new melilite-type borogermanates, Ca_2_GeB_2_O_7_ and Ca_1.78_Cd_0.22_GeB_2_O_7_, were also reported, among which Ca_2_GeB_2_O_7_ has a short UV cutoff edge (<200 nm), indicating its potential as an optical material in the UV or DUV region.^16^ The compounds mentioned above are the only borates with melilite structure reported so far. In contrast to many investigations on Bi_2_ZnB_2_O_7_, there are no studies of CaBiGaB_2_O_7_ and CdBiGaB_2_O_7_ as promising hosts for luminescence applications, and also, there are no reports on rare-earth analogues of these borates in the literature.

It is well known that Tb^3+^ ions usually generate green emission arising from the ^5^D_4_ → ^7^F_*J*_ (*J* = 6, 5, 4, 3) transitions, while Sm^3+^ ions emit orange-red light due to the ^4^G_5/2_ → ^6^H_*J*_ (*J* = 5/2, 7/2, 9/2, 11/2) transitions.^17^ In Tb^3+^/Sm^3+^ co-doped systems, terbium plays the role of a sensitizer, and samarium is an activator. The energy transfer from Tb^3+^ to Sm^3+^ occurs, which makes it possible to achieve the multicolor tunable luminescence from green to orange-red by simply adjusting the ratio of these two ions. With these materials, it will be more convenient to control the color output according to the practical application requirements. Therefore, the study of Tb^3+^/Sm^3+^ co-doped phosphors is not only of theoretical but also of practical significance, and some previously reported examples are KBaY(MoO_4_)_3_:Ln^3+^ (Ln^3+^ = Tb^3+^, Eu^3+^, Sm^3+^, Tb^3+^/Eu^3+^, Tb^3+^/Sm^3+^), CaLa_2_(MoO_4_)_4_ : Tb^3+^/Sm^3+^, and Ba_3_La(PO_4_)_3_ : Tb^3+^/Sm^3+^.^18–20^

In the process of exploring new borate materials to study their structure–property relationships, we found that Bi^3+^ in CdBiGaB_2_O_7_ can be completely replaced by Tb^3+^, resulting in a new melilite diborate, CdTbGaB_2_O_7_. Insofar as we know, it represents the first quaternary compound within CdO–Ln_2_O_3_–Ga_2_O_3_–B_2_O_3_ (Ln = trivalent rare-earth cations) system, and it is also the only rare-earth borate of the melilite family known to date. In this work, we first performed the synthesis and characterization of CdTbGaB_2_O_7_, then introduced Sm^3+^ into CdTbGaB_2_O_7_ to prepare CdTb_1−*x*_Sm_*x*_GaB_2_O_7_ solid solutions (*x* = 0–0.2), and further studied the luminescence properties and energy transfer of Sm^3+^ doped CdTbGaB_2_O_7_. The obtained results suggest that this type of novel phosphors could serve as a multi-color component in UV w-LEDs.

## 2. Experimental section

### 2.1. Materials and methods

All chemicals, including CdCO_3_ (A.R.), Tb_4_O_7_ (99.99%), Tb(NO_3_)_3_·6H_2_O (A.R.), Sm_2_O_3_ (99.99%), Ga_2_O_3_ (99.99%) and H_3_BO_3_ (A.R.), were commercially available from Sinopharm Chemical Reagent Co. Ltd and used without further purification. The XRD plots were recorded on a Bruker AXS D8 ADVANCE diffractometer equipped with Cu K_α1_ radiation (*λ* = 1.5406 Å) operating at 40 kV and 40 mA. Surface morphology and element compositions of the synthetic product were characterized by a Hitachi SU8020 field emission scanning electron microscope (FE-SEM) equipped with an energy dispersive X-ray spectrometer (EDX). The Infrared (IR) spectra were measured with a Bruker VERTEX70 FT-IR spectrometer using the KBr pellet method. The Raman studies were carried out using a Renishaw InVia Raman spectrometer equipped with a confocal DM 2500 Leica optical microscope, a thermoelectrically cooled CCD as a detector, and He/Ne laser as an exciting source working at 633 nm. The X-ray photoelectron spectroscopy (XPS) measurements were done by a Thermo ESCALAB 250xi spectrometer equipped with Al Kα (*hν* = 1486.6 eV) as an excitation source. The UV-vis absorption spectra were monitored using a Hitachi UH4150 spectrophotometer equipped with an integrating sphere attachment. The excitation and emission spectra as well as decay kinetics were investigated using an Edinburgh FLS 1000 system equipped with a 450 W Xe lamp and a 60 W μF flash lamp. Quantum yield (QY) and temperature-dependent emission spectra were determined by the same spectrometer with a BaSO_4_-coated integrating sphere and a temperature controlling system, respectively.

### 2.2. Synthetic procedures

Single crystals of CdTbGaB_2_O_7_ were grown by the high-temperature solution method. In a typical procedure, a powder mixture of 0.5464 g CdCO_3_, 0.1974g Tb_4_O_7_, 0.4950 g Ga_2_O_3_ and 0.2612 g H_3_BO_3_ (molar ratio 12 : 1 : 10 : 16) was thoroughly ground and placed in a Pt crucible. The mixture was slowly heated to 950 °C in a muffle furnace, and maintained at this temperature for 6 h to ensure that the raw materials are completely melted and uniformly mixed. Subsequently, the solution temperature was decreased, first to 700 °C at a rate of 1.5 °C h^−1^, then to 400 °C at 5.0 °C h^−1^, and finally to room temperature at 20 °C h^−1^. Many colorless, transparent, block-shaped crystals were obtained. Energy-dispersive X-ray analysis in a scanning electron microscope confirmed that the crystal contains the elements Cd, Tb, Ga, and O with an approximate atomic ratio of 10.44 : 8.25 : 6.65 : 74.66, which is close to its ideal composition of 1 : 1 : 1 : 7 (B is too light to be detected, see Fig. S1†).

The powder samples of CdTb_1−*x*_Sm_*x*_GaB_2_O_7_ (*x* = 0, 0.001, 0.002, 0.005, 0.01, 0.05, 0.1, 0.2, and 1) were obtained through solid-state reactions of the calculated amounts of CdCO_3_, Tb(NO_3_)_3_·6H_2_O, Sm_2_O_3_, Ga_2_O_3_, and H_3_BO_3_. The well-ground samples were first preheated at 500 °C for 12 h and then sintered at 800 °C for 120 h with several intermediate re-mixings. Finally, the as-synthesized samples were ground into fine powder and their phase purity was checked by powder X-ray diffraction.

### 2.3. Single-crystal X-ray diffraction

Single-crystal diffraction data were collected at room temperature on an Agilent Super Nova diffractometer equipped with a Mo X-ray source (*λ* = 0.71073 Å). The data collection and reduction were performed with the CrysAlisPro software, and absorption corrections were made by the multi-scan method.^21^ The crystal structure was established by Direct Methods and subsequently refined by the full-matrix least-squares method on *F*^2^ on the basis of SHELX-2018.^22^ All atoms were refined anisotropically and the final difference Fourier synthesis did not reveal any significant residual peaks.

Refinements of atomic occupancy parameters indicated that Cd and Tb atoms reside in the same atomic site (Wyckoff 4e) with the composition Cd_0.5_Tb_0.5_, which is not surprising since Cd^2+^ and Tb^3+^ have similar cationic radii (1.10 Å for Cd^2+^*vs.* 1.04 Å for Tb^3+^, CN = 8) and coordination geometries.^23^ For this (Cd/Tb) site, no abnormally large displacement parameters were observed, and the largest principal anisotropic displacement parameter [U_11_ = U_22_ = 0.01079(11) Å^2^] was only about 1.3 times of the smallest one [U_33_ = 0.00832(13) Å^2^] (Table S2†). Therefore, it is not necessary to split (Cd/Tb) into two positions. In addition, the single-crystal XRD data did not show a symmetry lower than tetragonal or a larger unit cell that would allow the ordering of Cd^2+^/Tb^3+^. Finally, the disorder model was adopted. The flack parameter of this compound was refined to be −0.12(4). The program PLATON was used to check the positional parameters,^24^ and no higher symmetries were found. Details of unit-cell parameters, data collection and structure refinements are summarized in Table 1. Atomic coordinates and equivalent isotropic and anisotropic displacement parameters are given in Tables S1 and S2† and selected bond lengths and angles in Table S3.†

**Table 1:** Crystallographic data for CdTbGaB_2_O_7_^a^

Formula	CdTbGaB_2_O_7_
Formula weight	474.66
Space group	*P*4̄2_1_*m* (No. 113)
*a* (Å)	7.3487(1)
*c* (Å)	4.7247(1)
*V* (Å^3^)	255.150(9)
*Z*	2
*d*_calc_ (g cm^−3^)	6.178
*μ* (mm^−1^)	23.062
2*θ*_max_ (°)	69.94
Unique reflections	620
Observed [*I* ≥ 2*σ*(*I*)]	598
No. of variables	35
GOF on *F*_o_^2^	1.092
*R*_1_/w*R*_2_ [*I* ≥ 2*σ*(*I*)]	0.0221/0.0443
*R*_1_/w*R*_2_ (all data)	0.0236/0.0449
Δ*ρ*_max_, Δ*ρ*_min_ (e Å^−3^)	1.528, −1.379

a *R*_1_ = Σ||*F*_o_| − |*F*_c_||/Σ|*F*_o_| and w*R*_2_ = [Σw(*F*_o_^2^ − *F*_c_^2^)^2^/Σw*F*_o_^4^]^1/2^ for *F*_o_^2^ > 2*σ*(*F*_o_^2^).

## 3. Results and discussion

### 3.1. Crystal structure description

**Fig. 1:**
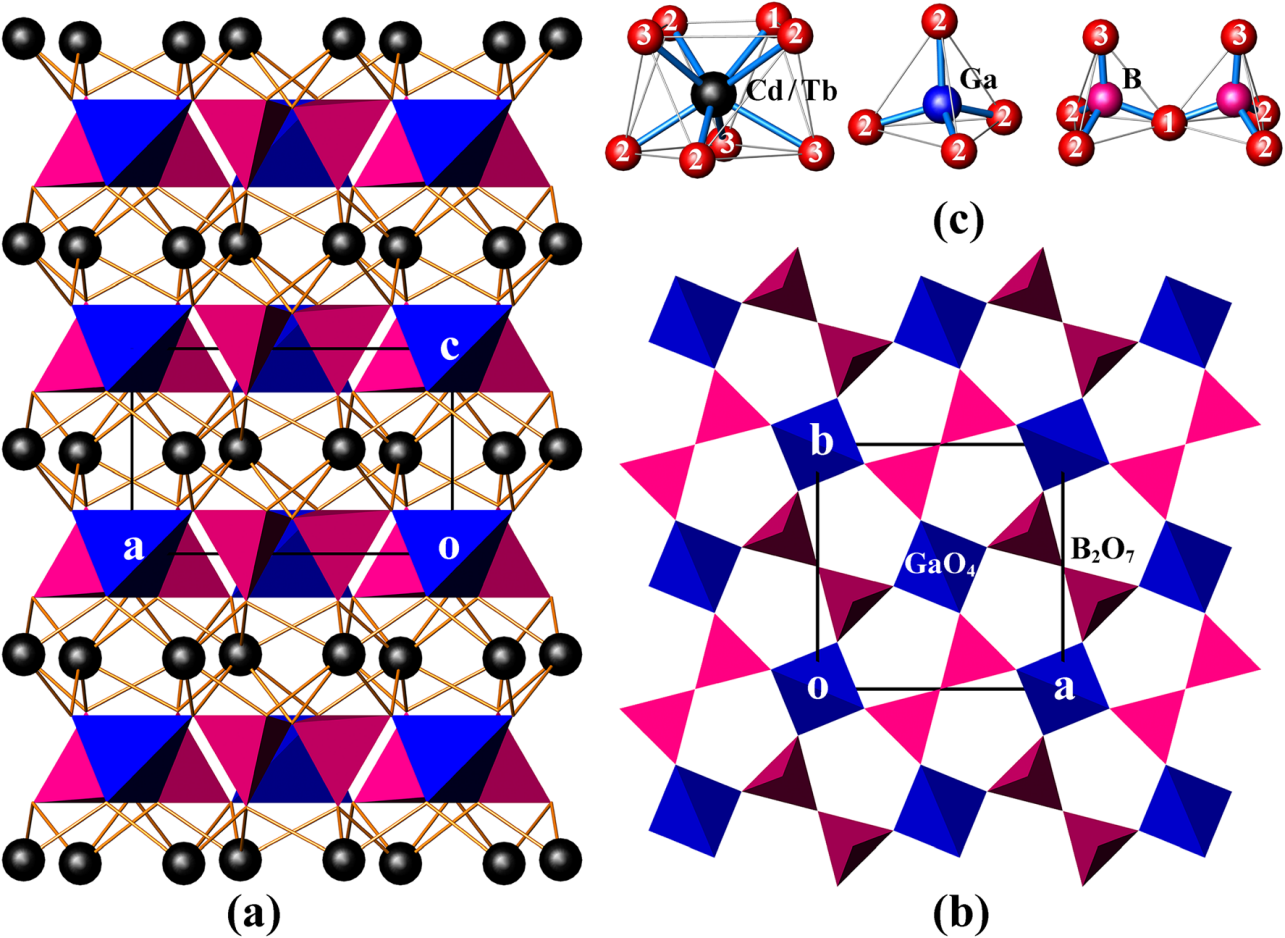
The unit cell of CdTbGaB_2_O_7_ projected along [010] (a), the [Ga(B_2_O_7_)]_*n*_^5*n*−^ layer viewed along [001] (b), and the coordination environment of each cation site (the numbers correspond to the oxygen atom designations) (c). (Cd/Tb): black spheres; GaO_4_: blue tetrahedra; BO_4_: magenta tetrahedra.

CdTbGaB_2_O_7_ is a new member of the melilite series, and it is also the only rare-earth borate of this family known so far. In this structure, two BO_4_ tetrahedra first share a corner to form a diborate group, [B_2_O_7_]^8−^. Each [B_2_O_7_]^8−^ dimer is linked to four different GaO_4_ tetrahedra, and, likewise, each GaO_4_ tetrahedron is connected to four neighboring [B_2_O_7_]^8−^ groups *via* common O atoms to form a 2D infinite [Ga(B_2_O_7_)]_*n*_^5*n*−^ layer extending in the (001) plane [see Fig. 1(b)]. These gallium borate anionic layers are stacked along the crystallographic *c*-axis direction, and (Cd^2+^/Tb^3+^) cations are placed in the square antiprismatic cavities between the layers to hold them together *via* (Cd/Tb)–O bonds, thus obtaining a 3D [CdTbGa(B_2_O_7_)]_*n*_ framework [Fig. 1(a)].

As shown in Table S1,† the asymmetric unit of CdTbGaB_2_O_7_ contains one disordered (Cd/Tb), one Ga, one B and three O sites. Among them, each (Cd/Tb) has eight O nearest-neighbors arranged into a distorted square antiprism [Fig. 1(c)]. The (Cd/Tb)–O distances fall in the range of 2.305(4)–2.547(3) Å, with an average of 2.430 Å (Table S3†), which lies between 2.42 and 2.48 Å computed from crystal radii sums of Tb^3+^ and O^2−^ as well as Cd^2+^ and O^2−^ for 8-fold coordination, respectively.^23^ These distances also correspond to those found in α-CdUO_4_, CdPd_3_O_4_ and Ba_2_CdTb_2_(BO_3_)_4_, where 8-coordinated Cd^2+^ or Tb^3+^ exist.^25–27^ Both Ga and B atoms adopt a tetrahedral coordination configuration. However, the Ga atom is located on the 4̄ axis, thereby resulting in four equal Ga–O bond lengths of 1.824(3) Å and two groups of O–Ga–O bond angles of 107.90(10)° and 112.7(2)°, while the B atom lies on a mirror plane, giving three groups of B–O distances of 1.419(7), 1.492(7) and 1.525(5) Å and four groups of O–B–O angles of 114.6(5)°, 116.1(3)°, 102.6(3)° and 102.9(4)°. The average O–Ga–O and O–B–O angles are 109.5° and 109.15°, respectively, indicating that the GaO_4_ tetrahedron is rather regular, while the BO_4_ is somewhat deformed (Table S3†). Similar GaO_4_ and BO_4_ groups have already been identified in K_2_Ga_2_O(BO_3_)_2_ and In_4_O_2_B_2_O_7_, respectively.^28,29^ Furthermore, the calculated bond valence sums are 3.10 for Ga^3+^ and 2.92 for B^3+^, respectively, close to their expected values, indicating the validity of the structure.^30^

In the literature, several compounds with the chemical formula containing “B_2_O_7_” are known, which have various structure types from extended 2D layers to 3D frameworks. For example, all Bi_2_CaB_2_O_7_, Bi_2_SrB_2_O_7_ and Bi_1.48_Eu_0.52_Pb_0.5_Sr_0.5_B_2_O_7_ contain topologically identical 2D [A_2_MO(BO_3_)_2_] [A = Bi, (Bi/Eu); M = Ca, Sr, (Pb/Sr)] layers built of corner-sharing [BO_3_]^3−^ triangles and [MO_6_]^10−^ trigonal prisms with [A_2_O]^4+^ groups accommodated within six-membered rings. However, the Ca and Sr compounds take an acentric structure with space groups *Pna*2_1_ and *P*6_3_, respectively, while the (Pb/Sr) phase crystallizes in the centrosymmetric *R*3̄*c* group.^31,32^ BaAl_2_B_2_O_7_ also has a layered structure, but being built up from [BO_3_]^3−^ triangles and [Al_2_O_7_]^8−^ groups, and the latter consists of two corner-sharing AlO_4_ tetrahedra.^33^ Bi_2_ZnB_2_O_7_ contains both tetrahedral [B_2_O_7_]^8−^ and triangular [B_2_O_5_]^4−^ diborate groups, which are alternately arranged in the *a* and *b* directions, and further bridged by tetrahedral Zn^2+^ centers through sharing three O atoms of each ZnO_4_ tetrahedron to generate a 2D [Zn_2_O_2_(B_2_O_7_)(B_2_O_5_)]_*n*_^12*n*−^ layer. These zinc borate anionic layers are held together by the octahedrally coordinated Bi^3+^ cations to create a 3D framework.^8^ This is different from the case of CdTbGaB_2_O_7_, where the 3D network is only composed of [B_2_O_7_]^8−^ tetrahedral dimers, GaO_4_ tetrahedra, and 8-coordinated (Cd^2+^/Tb^3+^) cations, and each GaO_4_ corner-shares with four [B_2_O_7_]^8−^ units. It is the differences in the fundamental building blocks and their connection modes that make these structures significantly different, which enriches the structural chemistry of borates.

### 3.2. IR and Raman spectra

CdLnGaB_2_O_7_ (Ln = Tb, Sm) crystallizes in the tetragonal space group *P*4̄2_1_*m* (D_2d_^3^, No. 113), and its primitive cell comprises two B_2_O_7_ “pyro” units. A free B_2_O_7_ group is composed of two corner-shared BO_4_ tetrahedra, which can be described as two BO_3_ groups connected by a bent B^O^B bridge, as shown in Fig. 1(c). In analogy with pyrophosphate and pyrogermanate groups (P_2_O_7_ and Ge_2_O_7_),^34–36^ the 21 internal modes of the free B_2_O_7_ group with *C*_2v_ symmetry can be subdivided into: A_1_ + B_1_ symmetric and A_1_ + A_2_ + B_1_ + B_2_ antisymmetric stretching modes of the BO_3_ groups [*ν*_s_(BO_3_) and *ν*_as_(BO_3_), respectively]; A_1_ symmetric and B_1_ antisymmetric stretching modes of the B^O^B bridge [*ν*_s_(B^O^B) and *ν*_as_(B^O^B), respectively]: A_1_ bending mode of the B^O^B bridge [*δ*(B^O^B)]; A_2_ + B_2_ rocking modes of the BO_3_ groups [*ρ*(BO_3_)]; and 3A_1_ + 2A_2_ + 3B_1_ + 2B_2_ O–B–O bending modes [*δ*(BO_3_)] (see the correlation diagram presented in Table S4†). In the crystal, these modes will give rise to 7A_1_ + 4A_2_ + 4B_1_ + 7B_2_ + 10E internal modes. Translational (T′) and librational (L) modes of free B_2_O_7_, *i.e.* A_1_ + B_1_ + B_2_ and A_2_ + B_1_ + B_2_, transform in crystal into A_1_ + B_2_ + 2E and A_2_ + B_1_ + 2E external modes, respectively. In addition, the disordered (Cd/Tb) atoms occupy the 4e Wyckoff positions of *C*_s_ symmetry and Ga atoms the 2a positions of S_4_ symmetry (Table S1†). These atoms contribute totally with 2A_1_ + A_2_ + 2B_1_ + 3B_2_ + 5E translational modes. By adding all these modes and subtracting three acoustic modes (B_2_ + E), the following optical vibrational modes of the crystal can be obtained: *Γ*_optic_ = 10A_1_ + 6A_2_ + 7B_1_ + 10B_2_ + 18E, in which E modes are twofold degenerated and often observed as one frequency. Among these modes, B_2_ and E are both IR- and Raman-active, A_1_ and B_1_ are Raman-active only, and A_2_ is silent. This analysis shows that 63 modes in the Raman spectrum (10A_1_ + 7B_1_ + 10B_2_ + 18E) and 46 modes in the IR spectrum (10B_2_ + 18E) are expected to be observed, resulting in 45 Raman and 28 IR frequencies, respectively. Due to the large number of modes and the overlap of some modes, a precise assignment of the individual bands to specific vibrational modes is difficult, but a rough assignment of groups is possible for both IR and Raman spectra.

Fig. S2† shows the room-temperature IR and Raman spectra of the CdLnGaB_2_O_7_ (Ln = Tb, Sm) samples. Due to experimental limitations, IR characterization below 500 cm^−1^ is not possible. Two compounds exhibit similar spectral profiles, reflecting their isostructural features. The tentative band assignments are based on literature data,^29,37–39^ and the frequencies of the B_2_O_7_ group are assigned according to the characteristic vibrations of the B^O^B bridge and the terminal BO_3_ groups. The bands due to the antisymmetric BO_3_ terminal stretching vibrations of the B_2_O_7_ group [*ν*_as_(BO_3_)] and the antisymmetric B^O^B bridge stretching modes [*ν*_as_(B^O^B)] are observed in the high-frequency region of 1250–900 cm^−1^. The intensities of these bands are generally greater in infrared than in Raman spectra.^38^ The strong Raman bands at about 787 (795) cm^− 1^ are assigned to the symmetric stretching of the terminal BO_3_ groups [*ν*_s_(BO_3_)], while in infrared spectra, these vibrations are clearly observed in the region of 800–840 cm^−1^. The IR bands near 704 (719) cm^−1^ can be attributed to the stretching vibrations of GaO_4_ tetrahedra,^40,41^ which matches with the peaks at 668 (676) cm^−1^ in the Raman spectra. The absorption bands at 600–300 cm^−1^ correspond to the *δ*(B^O^B) and *δ*(BO_3_) bending modes, and the bending vibrations of the GaO_4_ tetrahedra also appear in this area, making it difficult to assign the bands below 600 cm^−1^.^39,42^ Thus, the IR and Raman spectra confirm the presence of B_2_O_7_ tetrahedral dimers and four-coordinated Ga^3+^ ions, in accordance with the results obtained from the single-crystal XRD analyses.

### 3.3. UV-vis absorption spectra

Fig. S3† shows the UV-visible absorption spectra of CdLnGaB_2_O_7_ (Ln = Tb, Sm) (converted from diffuse reflectance data). The spectrum of the Tb compound shows a wide absorption band superimposed with several weak absorption peaks below 500 nm, which are mainly attributed to the inter-configurational 4f–5d transitions and intra-configurational 4f–4f transitions of the Tb^3+^ ions, i. e. ^7^F_6_ → ^5^L_8_ at 352 nm, ^7^F_6_ → ^5^L_10_ at 370 nm, ^7^F_6_ → ^5^D_3_ at 377 nm and ^7^F_6_ → ^5^D_4_ at 489 nm, respectively.^43^ For CdSmGaB_2_O_7_, there is a strong absorption band below 300 nm with maxima at about 224 nm, which is mainly ascribed to the ligand (O^2−^) to metal charge transfer (CT) transition. In the region between 300 and 500 nm, a series of sharp absorption peaks were observed at about 317, 346, 363, 377, 405, 418, 444, 465, 472, and 483 nm, which can be assigned to the ^6^H_5/2_ → ^4^F_11/2_, ^6^H_5/2_ → ^3^H_7/2_, ^6^H_5/2_ → ^4^F_9/2_, ^6^H_5/2_ → ^4^D_5/2_, ^6^H_5/2_ → ^4^K_11/2_, ^6^H_5/2_ → ^4^M_19/2_, ^6^H_5/2_ → ^4^G_9/2_ + ^4^I_15/2_, ^6^H_5/2_ → ^4^F_5/2_, ^6^H_5/2_ → ^4^I_11/2_, ^6^H_5/2_ → ^4^M_15/2_ transitions, respectively, as found in the PLE spectrum [Fig. 4(b)].^44,45^

The optical band gap (*E*_g_) can be estimated with the help of Tauc's relation:^46^*αhν* = *A*(*hν* − *E*_g_)^*n*^where *α*, *h*, *ν*, *A* and *E*_g_ stand for absorption coefficient, Planck's constant, light frequency, proportionality constant and band gap, respectively. n is a constant related to the transition (*n* = 1/2 and 2 for direct and indirect allowed transitions, respectively). If the absorption coefficient α is replaced by the absorbance obtained from the UV-vis spectrum, (*αhν*)^1/*n*^ is plotted against the photon energy (*hν*), and on this basis, a line tangent to the inflection point of the curve is further drawn, the intercept of the tangent to the *x*-axis will give a good approximation of the band gap energy. In the present case, the indirect and direct band gaps are estimated to be 4.10 and 4.33 eV for CdTbGaB_2_O_7_, whereas they are 3.82 (indirect) and 4.87 eV (direct) for CdSmGaB_2_O_7_, respectively, as shown in the insets of Fig. S3.† These bandgap values are similar to those previously reported in other Tb- and Sm-containing borates, such as K_3_Li_3_Tb_7_(BO_3_)_9_ [4.19 (indirect) and 4.55(direct) eV] and K_3_Sm_3_(BO_3_)_4_ [4.99 (direct) eV].^47,48^

### 3.4. Phase purity and morphology

**Fig. 2:**
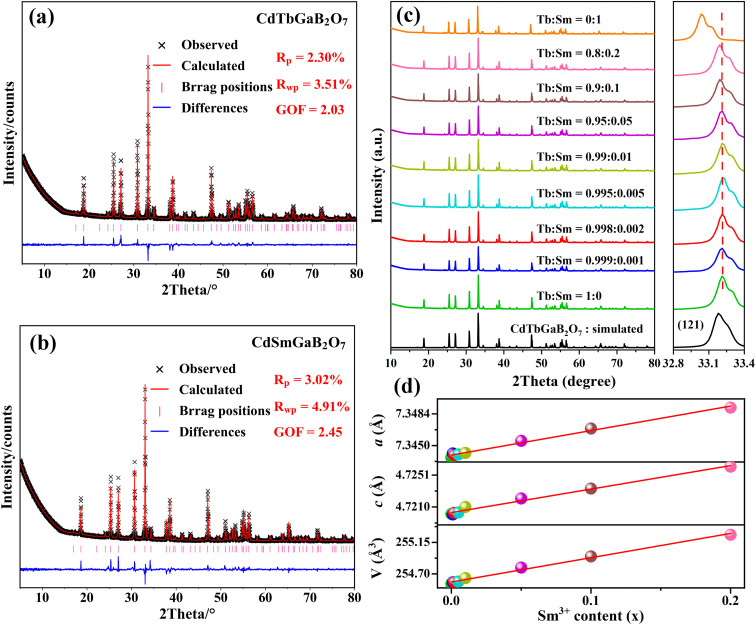
(a and b) Rietveld refinements of the XRD files for CdTb_1−*x*_Sm_*x*_GaB_2_O_7_ (*x* = 0 and 1). (c) XRD patterns of the CdTb_1−*x*_Sm_*x*_GaB_2_O_7_ (0 ≤ *x* ≤ 1) phosphors. (d) The cell parameters (*a*, *c*, and *V*) obtained by Rietveld refinements against the Sm^3+^ concentration (*x*).

In order to check the correctness of the single-crystal structure, additional Rietveld refinements of powder XRD data from CdTb_1−*x*_Sm_*x*_GaB_2_O_7_ (*x* = 0 and 1) were carried out using TOPAS software.^49^ In the refining process, the single-crystal data of the Tb phase were employed as the initial model, a total of 51 parameters were refined, including 47 profile and 4 structure ones. The final refinement patterns are depicted in Fig. 2(a) and (b), where the low *R*-factors and the small differences between the measured and calculated data were obtained, indicating good fitting quality and high phase purity. Although the cell dimensions of CdTbGaB_2_O_7_ from Rietveld refinements [*a* = *b* = 7.3437(1) Å, *c* = 4.7201(1) Å, and *V* = 254.558(8) Å^3^] are somewhat smaller than the single-crystal data [Table 1], their relative deviations are less than 0.5%, revealing that the determined structure should be quite credible.

Powder XRD patterns of the as-prepared CdTb_1−*x*_Sm_*x*_GaB_2_O_7_ (0 ≤ *x* ≤ 1) samples are presented in Fig. 2(c). All experimental diagrams agree well with the simulated pattern of CdTbGaB_2_O_7_, indicating that doping of Sm^3+^ ions did not generate any distinct impurity or induce significant changes in the host structure. In addition, the enlargement of the reflections in the range of 2*θ* = 32.8°–33.4° is shown in the right part of Fig. 2(c), where a continuous left-shift of the representative peak (121) can be clearly observed with increasing Sm^3+^ content in CdTb_1−*x*_Sm_*x*_GaB_2_O_7_. In fact, as we discussed earlier, Cd and Tb atoms in CdTbGaB_2_O_7_ are statistically distributed over one atomic site, and each (Cd/Tb) is surrounded by eight oxygen atoms, forming a distorted (Cd/Tb)O_8_ polyhedron, while Ga and B atoms are completely ordered and tetrahedrally coordinated with oxygen atoms. Considering the ionic radii and coordination number (CN) of Cd^2+^ (1.10 Å, CN = 8), Tb^3+^ (1.040 Å, CN = 8), Sm^3+^ (1.079 Å, CN = 8), Ga^3+^ (0.47 Å, CN = 4) and B^3+^ (0.11 Å, CN = 4),^23^ it is reasonable to believe that the doped Sm^3+^ ions will replace Tb^3+^ and occupy (Cd/Tb) sites randomly in the host, which will lead to lattice expansion, thus shifting the diffraction peaks to smaller 2*θ* values according to the Bragg equation.

The ionic radii for eightfold coordinated Tb^3+^ and Sm^3+^ differ only by 3.75% and both fully concentrated Tb^3+^ and Sm^3+^ compounds are isostructural. Therefore, it is expected that there is a solid solution series whose lattice constants are linearly related to the Sm^3+^/Tb^3+^ ratio. In order to confirm this point, Rietveld refinements of powder XRD profiles were also performed on CdTb_1−*x*_Sm_*x*_GaB_2_O_7_ (*x* = 0.001, 0.002, 0.005, 0.01, 0.05, 0.1 and 0.2), as shown in Fig. S4.† For these refinements, the atomic coordinates of Ga, B and O sites, atomic occupancies, and isotropic thermal displacement factors were fixed, while the atomic coordinates of the (Cd/Tb/Sm) site and cell parameters were refined along with other parameters. The lattice parameters and atomic coordinates of all species obtained from Rietveld fitting are summarized in Tables S5 and S6,† while the lattice parameters for 0 ≤ *x* ≤ 0.2 were plotted as a function of the dopant Sm^3+^ concentration (*x*) in Fig. 2(d). Obviously, cell parameters, including axis lengths (*a* and *c*) and volume (*V*), increase linearly with the incorporation of Sm^3+^, as predicted by Vegard law. This phenomenon can be explained by the larger ionic radius of Sm^3+^ compared to that of Tb^3+^, which is strong evidence of the successful cationic substitution.

**Fig. 3:**
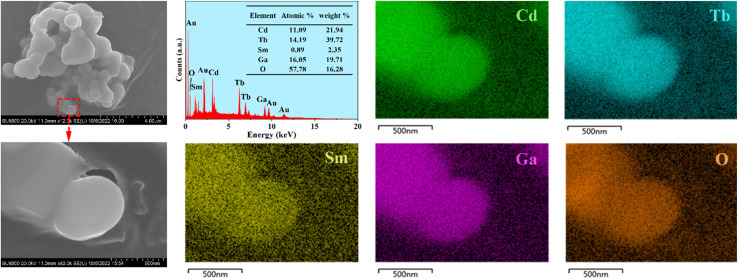
FE-SEM images, typical EDS results, and elemental mapping of the CdTb_0.995_Sm_0.005_GaB_2_O_7_ phosphor. Note that a thin layer of Au was evaporated on the sample surface to provide conductivity prior to SEM inspection.

The SEM images of the representative phosphor CdTb_0.995_Sm_0.005_GaB_2_O_7_ are illustrated in Fig. 3. The particles exhibit irregular shapes in the agglomerated form with several microns in size. They are typical products prepared by the high-temperature solid state reaction. Besides, the EDX spectrum reveals that in addition to Au as a coating element, there are expected elements in the studied sample, including Cd, Tb, Sm, Ga and O (B is too light to be detected). The elemental mappings demonstrate that all constituent elements are evenly distributed among the selected particle. Hence, the successful doping of Sm^3+^ into the CdTbGaB_2_O_7_ matrix to form a homogenous phase can be verified based on the XRD and EDX results.

### 3.5. XPS spectra

XPS was performed to determine the surface chemical components of the phosphor CdTb_0.995_Sm_0.005_GaB_2_O_7_. Fig. S5(a)† depicts a typical survey scan in the range of 1350–0 eV, which contains Cd 3p, 3d, 4d, Tb 3d, 4d, Ga 2p, 3d, B 1s, O 1s and C 1s core levels along with Ga and O Auger peaks, confirming the sample composition. The presence of C can be ascribed to the expected adsorption of adventitious carbon from XPS instrument itself, and the C 1s peak of 284.6 eV was used as a standard to adjust other peaks. To further inspect the chemical state of each element, narrow scan XPS measurements were done, and the results are shown in Fig. S5(b)–(g).† One can see that there are two distinct peaks at 411.62 and 404.79 eV in Fig. S5(b),† which are assigned to Cd 3d_3/2_ and 3d_5/2_ core levels, and can be used as a fingerprint to identify the existence of Cd^2+^.^50^ The Tb 3d XPS spectrum is depicted in Fig. S5(c),† where two strong peaks associated with Tb 3d_3/2_ and 3d_5/2_ were clearly observed at 1278.71 and 1243.76 eV. These binding energies and their differences confirm the +3 valence state of Tb in the doped system.^51^ In Fig. S5(d),† although the Sm 4d XPS signal is relatively weak because of the very low dopant concentration in the phosphor, it can be deconvoluted into three components, corresponding to Sm 4d_3/2_ (∼134.29 eV), 4d_5/2_ (∼130.98 eV) and a satellite peak (127.87 eV), respectively, similar to the earlier results obtained for β-RbSm(MoO_4_)_2_.^52^ This indicates that the doped Sm element predominantly maintains the state of Sm^3+^ in the CdTbGaB_2_O_7_ matrix. Furthermore, the Ga 3p spectrum is composed of doublet components at 108.58 eV (Ga 3p_1/2_) and 104.96 eV (Ga 3p_3/2_) with the spin–orbit splitting of ∼3.62 eV, indicating that Ga appears as +3 valence.^53^ The B 1s spectrum was fitted to a single peak, which is attributed to the B atoms bonded to oxygen atoms (B–O) in borates, and its binding energy of 191.57 eV is in good accord with the literature report.^54^ The O 1s spectrum can be resolved into two components, of which the main peak at 530.95 eV usually denotes the presence of structural oxide (O^2−^), while the shoulder peak at 532.77 eV corresponds to the oxygen in adsorbed water (H_2_O) at the sample surface.^55^ No other impurity peaks have been identified from the XPS spectra, supporting the aforementioned XRD and EDX analyses. All these observations ensure the successful cationic substitution, after which a systematic investigation of the photoluminescence properties of the CdTb_1−*x*_Sm_*x*_GaB_2_O_7_ phosphors was conducted.

### 3.6. PLE and PL spectra

**Fig. 4:**
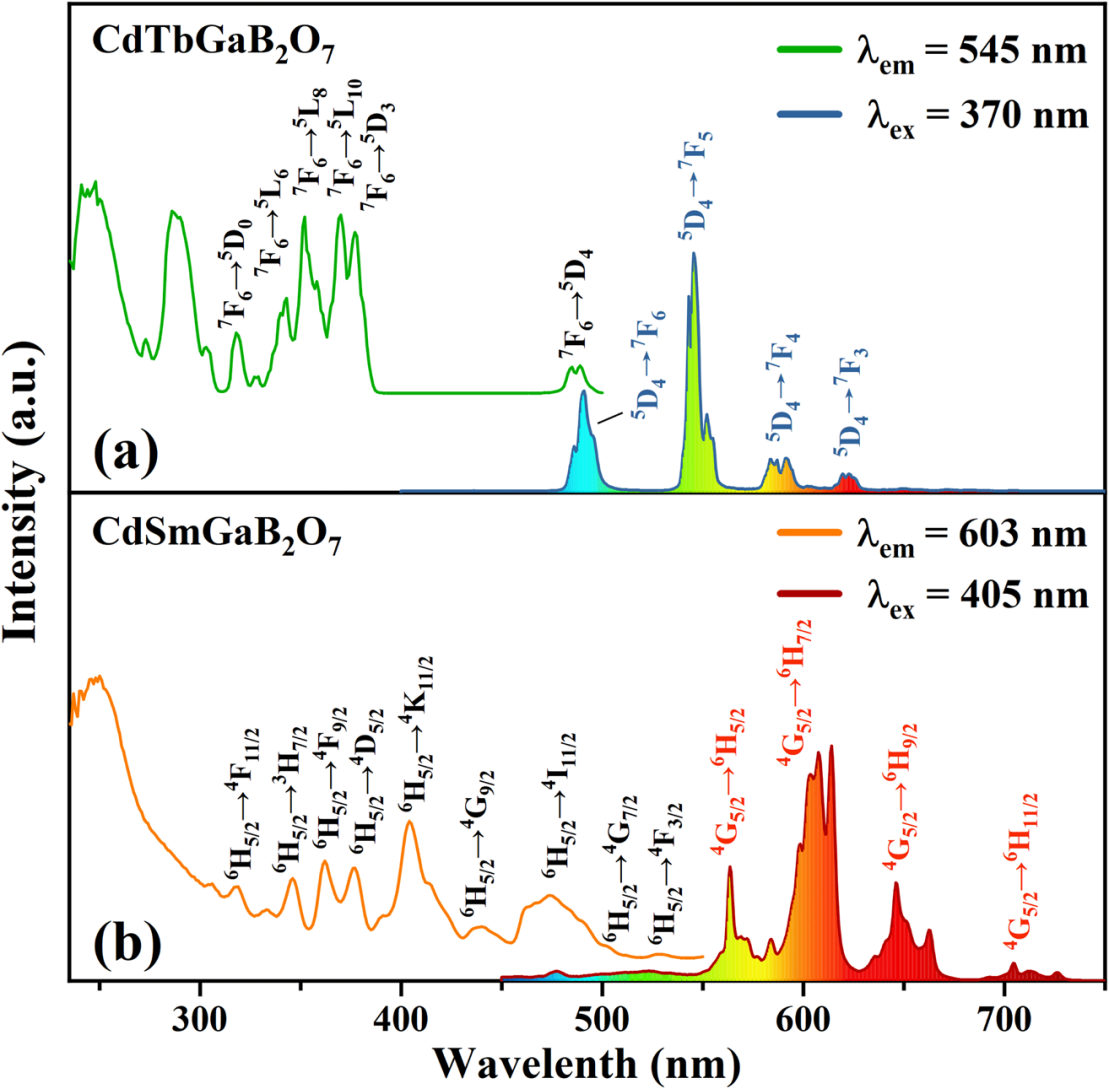
The PLE and PL spectra of CdTbGaB_2_O_7_ (a) and CdSmGaB_2_O_7_ (b).

Fig. 4(a) shows the typical photoluminescence excitation (PLE) and emission (PL) spectra of the CdTbGaB_2_O_7_ phosphor. The PLE spectrum obtained by monitoring 545 nm emission (Tb^3+^: ^5^D_4_ → ^7^F_5_) consists of two strong broad bands below 300 nm, along with many sharp peaks in the higher wavelength region. The strong broadband centered at ∼248 and 286 nm can be attributed to the spin-allowed (^7^F_6_–^7^D_*J*_) and spin-forbidden (^7^F_6_–^9^D_*J*_) 4f^8^ → 4f^7^5d^1^ inter-configurational Tb^3+^ transitions, respectively.^56^ The other excitation peaks appearing at 318, 343, 352, 370, 377, and 485 nm correspond to the parity- and spin-forbidden 4f–4f transitions of the Tb^3+^ ion, that is, from the ^7^F_6_ ground state to the excited states of ^5^D_0_, ^5^L_6_, ^5^L_8_, ^5^L_10_, ^5^D_3_, and ^5^D_4_ levels, as labelled in Fig. 4(a).^43^ The PL spectrum recorded under the excitation of 370 nm gives four sets of characteristic optical transitions of Tb^3+^, namely ^5^D_4_ → ^7^F_*J*_ (*J* = 6, 5, 4, and 3) at approximately 491, 545, 587 and 623 nm, respectively. The transition of ^5^D_4_ → ^7^F_5_ at ∼545 nm is the most prominent, which explains the strong green emission of CdTbGaB_2_O_7_. As expected, no emission coming from ^5^D_3_ level has been observed due to the fast cross relaxation processes [Tb^3+^ (^5^D_3_) + Tb^3+^ (^7^F_6_) → Tb^3+^ (^5^D_4_) + Tb^3+^ (^7^F_0_)] present in fully concentrated terbium materials.^57^

The PLE and PL spectra of the CdSmGaB_2_O_7_ phosphor are presented in Fig. 4(b). The PLE spectrum monitored with 603 nm emission exhibits a broadband near 250 nm together with multiple sharp peaks at 319, 346, 362, 377, 405, 440, 474, 502 and 528 nm, which are associated with the O^2−^ → Sm^3+^ charge–transfer transition and the characteristic Sm^3+^ f–f transitions: ^6^H_5/2_ → ^4^F_11/2_, ^6^H_5/2_ → ^3^H_7/2_, ^6^H_5/2_ → ^4^F_9/2_, ^6^H_5/2_ → ^4^D_5/2_, ^6^H_5/2_ → ^4^K_11/2_, ^6^H_5/2_ → ^4^G_9/2_, ^6^H_5/2_ → ^4^I_11/2_, ^6^H_5/2_ → ^4^G_7/2_ and ^6^H_5/2_ → ^4^F_3/2_, respectively.^44,45^ The presence of the intense absorption bands from 350 to 490 nm suggests that CdSmGaB_2_O_7_ can be used as a potential phosphor for UV/NUV LED lighting. When the phosphor is exposed to 405 nm radiation, it emits luminescence at about 564, 603, 646, and 705 nm, assigned to the ^4^G_5/2_ → ^6^H_*J*_ (*J* = 5/2, 7/2, 9/2, 11/2) transitions of Sm^3+^, respectively. Among them, ^4^G_5/2_ → ^6^H_5/2_ and ^4^G_5/2_ → ^6^H_9/2_ are purely magnetic dipole (MD) and electric dipole (ED) allowed transitions, respectively, while ^4^G_5/2_ → ^6^H_7/2_ is a MD allowed one but also ED dominated. Generally speaking, the integral intensity ratio of the ED/MD transitions [R = I(^4^G_5/2_ → ^6^H_9/2_)/I(^4^G_5/2_ → ^6^H_5/2_)] is used to assess the symmetry nature of trivalent 4f ions. In this work, the R value was calculated as 1.45 (>1), implying that Sm^3+^ ions are located in the low symmetry sites. In fact, our structural analysis reveals that Sm^3+^ will replace Tb^3+^ and share the Wyckoff 4e site with Cd^2+^, which is on a mirror plane without inversion center, as shown in Table S1† and Fig. 1(c).

**Fig. 5:**
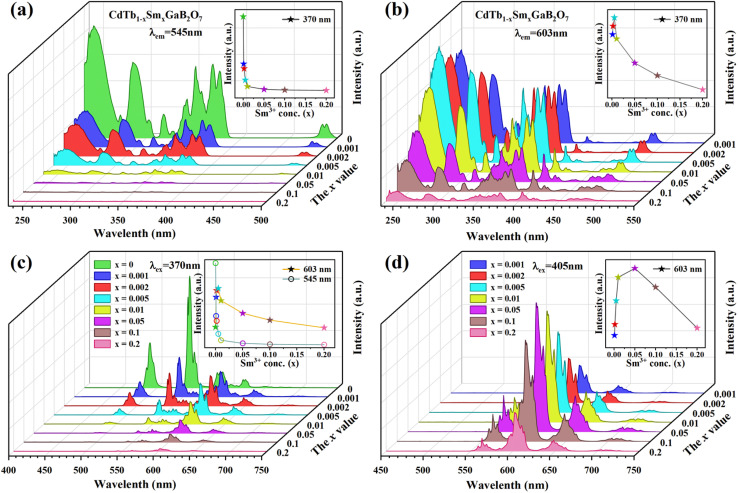
The concentration dependent PLE [(a) *λ*_em_ = 545 nm; (b) *λ*_em_ = 603 nm] and PL [(c) *λ*_e*x*_ = 370 nm; (d) *λ*_e*x*_ = 405 nm] spectra of the CdTb_1−*x*_Sm_*x*_GaB_2_O_7_ fluorescent powders.

The PLE spectra of the CdTb_1−*x*_Sm_*x*_GaB_2_O_7_ phosphors with different Sm^3+^ concentrations are shown in Fig. 5(a) and (b). By monitoring the Tb^3+^ emission at 545 nm (^5^D_4_ → ^7^F_5_), the PLE spectra show the f–d and f–f transitions of Tb^3+^ and the excitation intensity decreases remarkably and steadily towards a higher Sm^3+^ content and finally becomes negligible at *x* = 0.2, suggesting the presence of efficient Tb^3+^ → Sm^3+^ energy transfer. It is noteworthy that Sm^3+^ excitation peaks were undetectable in each case, which ascertains the fact that there was no possibility of energy transfer from Sm^3+^ to Tb^3+^. On the other hand, the PLE spectra monitored with the 603 nm emission (Sm^3+^: ^4^G_5/2_ → ^6^H_7/2_) contain not only the characteristic excitation peak of Sm^3+^ at 405 nm, but also those of Tb^3+^ originated from f–d and f–f transitions, with the latter dominating the PLE spectra, which further confirms the efficient Tb^3+^ → Sm^3+^ energy transfer. Besides, the intensities of the Tb^3+^ excitation transitions (*e.g.*, ^7^F_6_–^5^L_10_ at 370 nm) successively increase with increasing Sm^3+^ concentration up to *x* = 0.005 and then decrease, implying the occurrence of concentration quenching at *x* > 0.005.

The influence of Sm^3+^ content on the PL spectra of CdTb_1−*x*_Sm_*x*_GaB_2_O_7_ were also studied, as shown in Fig. 5(c) and (d). As expected, upon excitation at 370 nm (Tb^3+^: ^7^F_6_ → ^5^L_10_), CdTbGaB_2_O_7_ displays only the characteristic emissions of Tb^3+^. With the doping of Sm^3+^ ions, besides Tb^3+^ emissions at 491 (^5^D_4_ → ^7^F_6_) and 545 nm (^5^D_4_ → ^7^F_5_), the characteristic emissions of Sm^3+^ at 603 nm (^4^G_5/2_ → ^6^H_7/2_) and 646 nm (^4^G_5/2_ → ^6^H_9/2_) can also be observed. Furthermore, as the Sm^3+^ concentration increases, the emission intensity of Tb^3+^ at 545 nm first decreases rapidly, and then remains at a relative low value with little difference, as illustrated in the inset of Fig. 5(c). At the same time, the emission intensity of Sm^3+^ at 603 nm increases initially until the Sm^3+^ concentration reaches *x* = 0.005, reflecting the result of energy transfer from Tb^3+^ to Sm^3+^. However, once the Sm^3+^ content is further increased to beyond *x* = 0.005, the concentration quenching occurs between Sm^3+^ ions, resulting in a decrease in the emission intensity. In addition, the PL spectra under direct excitation of the Sm^3+^ 4f levels at 405 nm (Sm^3+^: ^6^H_5/2_ → ^4^K_11/2_) yield only the emission peaks of Sm^3+^ and not those of Tb^3+^, further confirming the absence of energy transfer from Sm^3+^ to Tb^3+^. In this case, the emission intensity of Sm^3+^ first increases until *x* = 0.05 and then appears a downfall, which is consistent with the trend of Sm^3+^ characteristic excitation peak at 405 nm in the PLE spectra monitored at 603 nm emission of Sm^3+^ [Fig. 5(b)]. However, it is different from the situation observed in the 370 nm indirect excitation *via* the energy transfer from Tb^3+^ to Sm^3+^, where the optimal Sm^3+^ doped content is *x* = 0.005. As everyone knows, the PL spectrum is strongly affected by the excitation wavelength.^58^ The fact that indirect and direct excitation leads to different quenching concentrations has already been reported in some other phosphors, such as Ba_3_BiPbY_1−*x*_Eu_*x*_O(BO_3_)_4_, CeO_2_ : Eu^3+^ and SnO_2_ : Eu^3+^.^59–61^

### 3.7. Fluorescence lifetime and energy transfer mechanism

**Fig. 6:**
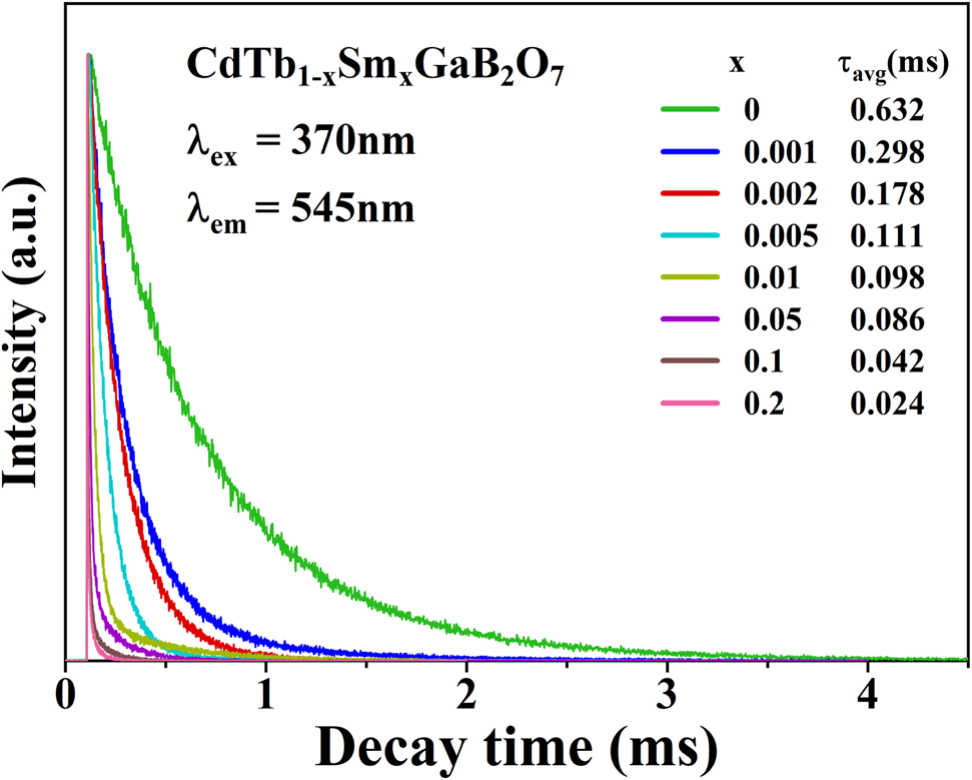
Tb^3+^ decay curves of the CdTb_1−*x*_Sm_*x*_GaB_2_O_7_ (0 ≤ *x* ≤ 0.2) phosphors monitoring 545 nm emission.

To further illustrate the Tb^3+^ → Sm^3+^ energy transfer process, the Tb^3+^ luminescence decay curves of the CdTb_1−*x*_Sm_*x*_GaB_2_O_7_ phosphors (*λ*_e*x*_ = 370 nm, *λ*_em_ = 545 nm) were recorded, as shown in Fig. 6. All curves show a second-order exponential decay, which can be fitted by the following equation:
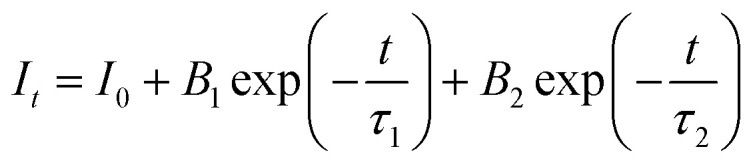
where *I*_0_ and *I*_*t*_ are the luminescence intensities corresponding to time 0 and *t*, *B*_1_ and *B*_2_ are fitting parameters, and *τ*_1_ and *τ*_2_ are lifetimes for rapid and slow decays, respectively. The double-exponential behavior indicates a heterogeneous distribution of Tb^3+^ ions in the CdTb_1−*x*_Sm_*x*_GaB_2_O_7_ phosphors. In fact, when the excitation energy is transferred from the donor (Tb^3+^) to acceptor (Sm^3+^), a double-exponential decay behavior of the activator is usually observed.^17^ The decay process is characterized by an average lifetime (*τ*_avg_), which can be calculated as follows:
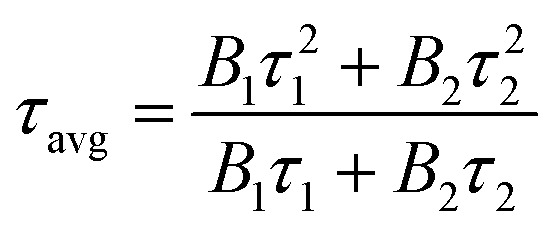


The *τ*_avg_ values determined for different Sm^3+^ concentrations are also shown in Fig. 6. Apparently, the luminescence lifetime of Tb^3+^ in CdTb_1−*x*_Sm_*x*_GaB_2_O_7_ decreases successively with the increase of Sm^3+^ concentration, which offers clear evidence for the energy transfer from Tb^3+^ to Sm^3+^. The energy transfer efficiency (*η*) from sensitizer to activator can be evaluated with the following expression:
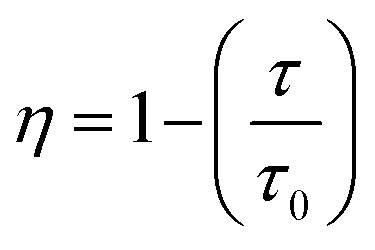
where τ and *τ*_0_ are the decay times of Tb^3+^ (^5^D_4_ → ^7^F_5_) with and without Sm^3+^ co-doping. The calculated *η* values are provided in Table 2. It is found that the energy transfer efficiency increases monotonously from 52.85% to 96.20% with the raising Sm^3+^ concentration from *x* = 0.001 to 0.2.

**Table 2:** CIE chromaticity coordinates, CCT values and energy transfer efficiencies (*η*) of the CdTb_1−*x*_Sm_*x*_GaB_2_O_7_ (0 ≤ *x* ≤ 0.2) phosphors

CdTb_1−*x*_Sm_*x*_GaB_2_O_7_	*λ*_e*x*_ (nm)	CIE (*x*, *y*)	CCT (K)	*η* (%)
1 *x* = 0	370	(0.3134, 0.5750)	5854	—
2 *x* = 0.001	370	(0.4384, 0.4957)	3574	52.85
3 *x* = 0.002	370	(0.4656, 0.4779)	3093	71.84
4 *x* = 0.005	370	(0.5213, 0.4430)	2227	82.44
5 *x* = 0.01	370	(0.5539, 0.4202)	1838	84.49
6 *x* = 0.05	370	(0.5739, 0.4044)	1677	86.39
7 *x* = 0.1	370	(0.5746, 0.3979)	1655	93.35
8 *x* = 0.2	370	(0.5391, 0.3798)	1706	96.20

**Fig. 7:**
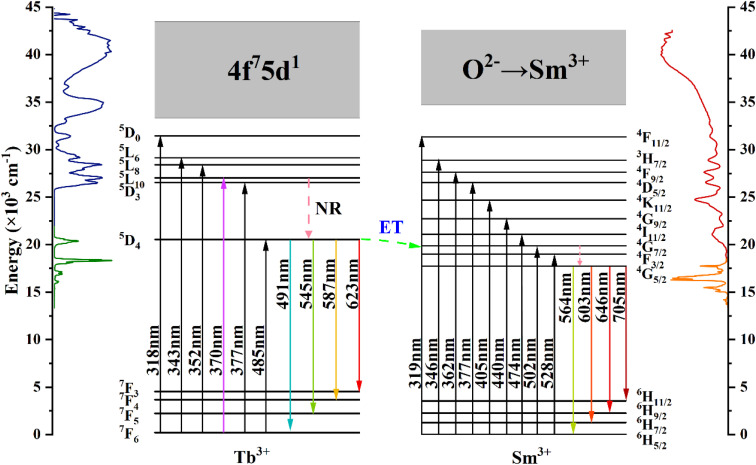
Schematic energy-level diagrams of Tb^3+^ and Sm^3+^ in CdTb_1−*x*_Sm_*x*_GaB_2_O_7_, showing energy-transfer process (ET: energy transfer; NR: nonradiative).

Fig. 7 shows the energy level diagrams of Tb^3+^ and Sm^3+^ ions illustrating the energy migration processes in CdTb_1−*x*_Sm_*x*_GaB_2_O_7_. Upon excitation at 370 nm, the electrons on Tb^3+^ ions can jump from the ^7^F_6_ ground state to the ^5^L_10_ excited state. Afterwards, the nonradiative transition (NR) took place, leading to the population of the ^5^D_4_ level. While some of the excited electrons return to the ^7^F_6,5,4,3_ ground state level in a radiative manner, generating the typical Tb^3+^ emissions. The remaining electrons at the ^5^D_4_ level of Tb^3+^ can move to the ^4^G_7/2_ excited level of Sm^3+^ due to energy level matching, followed by nonradiative relaxation to the ^4^G_5/2_ excited state and then radiative relaxation to the ^6^H_*J*_ (*J* = 5/2, 7/2, 9/2, 11/2) ground states to produce the observed Sm^3+^ emissions. This process enhances the characteristic emission of Sm^3+^ and simultaneously reduces the fluorescence emission intensity of Tb^3+^. In addition, it can be seen from Fig. 4(a) and (b) that there is a significant spectral overlap between the emission band of Tb^3+^ and the excitation band of Sm^3+^ in the range of 475–525 nm, which indicates that the energy transfer from Tb^3+^ to Sm^3+^ can be anticipated in CdTb_1−*x*_Sm_*x*_GaB_2_O_7_*via*^5^D_4_ channel: ^5^D_4_ (Tb^3+^) + ^6^H_5/2_ (Sm^3+^) → ^7^F_6_ (Tb^3+^) + ^4^I_11/2_ (Sm^3+^).^62^ The Tb^3+^ → Sm^3+^ energy transfer is almost irreversible because the ^5^D_4_ level of Tb^3+^ is slightly higher than the ^4^G_7/2_ level of Sm^3+^, which also explains the observation that Tb^3+^ cannot be excited by 405 nm (Sm^3+^:^6^H_5/2_ → ^4^K_11/2_ transition) in this system.

### 3.8. Emitting light color analysis

**Fig. 8:**
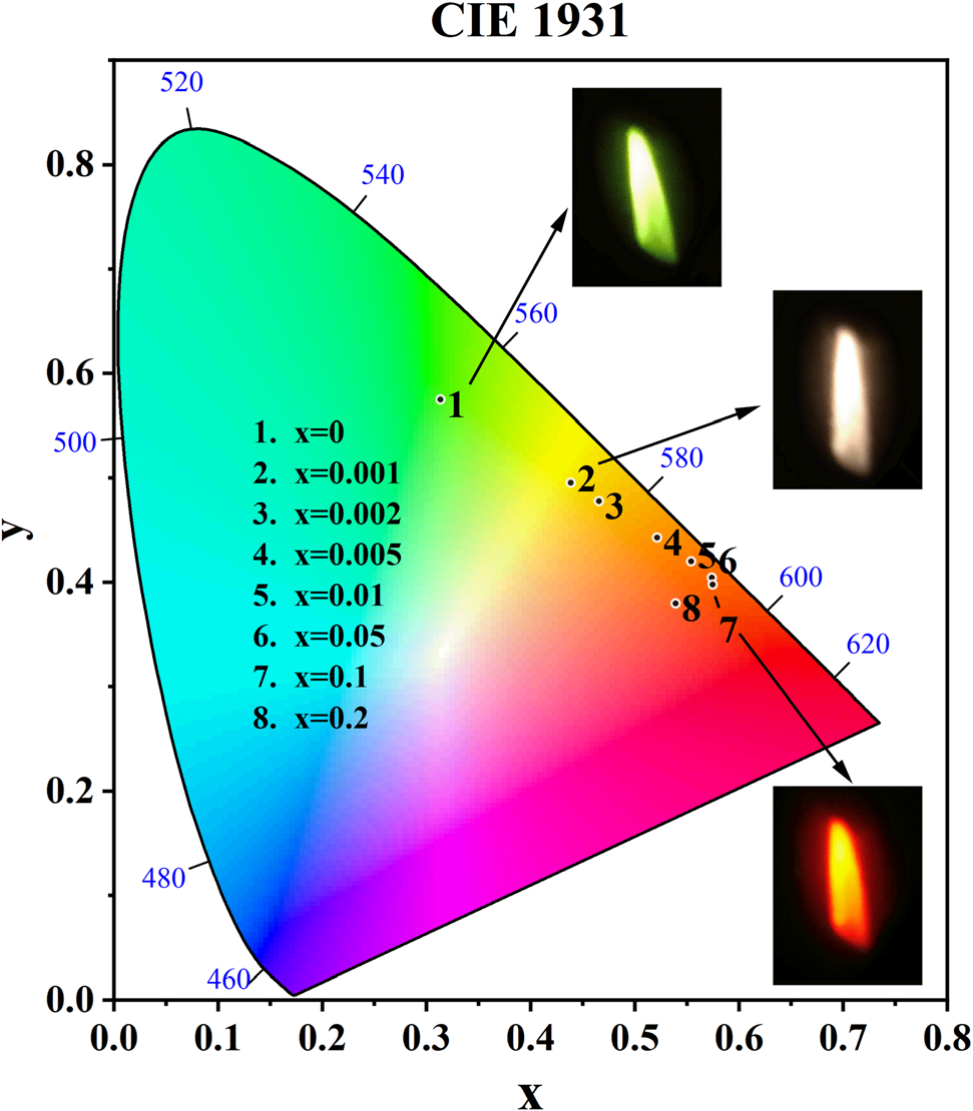
CIE chromaticity coordinates of the CdTb_1−*x*_Sm_*x*_GaB_2_O_7_ (*x* = 0, 0.001, 0.002, 0.005, 0.01, 0.05, 0.1, 0.2) phosphors and digital photographs of the selected samples (*x* = 0, 0.001 and 0.1) (*λ*_ex_ = 370 nm).

The CIE coordinates and CIE chromaticity diagram for the samples CdTb_1−*x*_Sm_*x*_GaB_2_O_7_ (0 ≤ *x* ≤ 0.2) were measured, as shown in Table 2 and Fig. 8. The inset of Fig. 8 also shows the digital photos of the selected phosphors under a 370 nm UV lamp. One can see that the CdTbGaB_2_O_7_ host emits green light when excited at 370 nm. Once Sm^3+^ ions were doped into this host and the Sm^3+^ content changed from *x* = 0 to 0.1, the CIE coordinates of CdTb_1−*x*_Sm_*x*_GaB_2_O_7_ would vary almost linearly from (0.3134, 0.5750) to (0.5746, 0.3979), which means that the emitting color changes continuously from green to orange-red. In addition, as seen from Fig. 5(c), when the Sm^3+^ content was further increased from *x* = 0.1 to 0.2, the characteristic blue and green emissions of Tb^3+^ remained basically unchanged, while the orange and red emissions of Sm^3+^ were significantly reduced due to the concentration quenching effect. Therefore, the chromaticity coordinates of the *x* = 0.2 sample are shifted toward the blue direction with respect to the *x* = 0.1 sample. A similar phenomenon was found in some previously reported Tb^3+^ and Sm^3+^ codoped phosphors, such as Y_3_Al_2_Ga_3_O_12_ : 0.5Tb^3+^, *y*Sm^3+^, Na_3_Bi(PO_4_)_2_ : 0.1Tb^3+^, *x*Sm^3+^ and LaAl_2.03_B_4_O_10.54_ : 0.1Tb^3+^, *y*Sm^3+^.^62–64^ Next is the CCT (the Correlated Color Temperature), which is a measure of how cool or warm the appearance of a light source will be.^65^ The CCT can be calculated by the analytical equation proposed by McCamy:^66^CCT = −449*n*^3^ + 3525*n*^2^ − 6823.3*n* + 5520.33where *n* = (*x* − *x*_e_)/(*y* − *y*_e_) and (*x*_e_ = 0.332, *y*_e_ = 0.186). The obtained CCT values for the CdTb_1−*x*_Sm_*x*_GaB_2_O_7_ (0.001 ≤ *x* ≤ 0.2) phosphors decrease with the enhancement of Sm^3+^ concentration and are in the range of 1655–3574 K (Table 2), which means that the prepared phosphors have the “neutral to warm” colors. The above results indicate that multicolor photoluminescence can be realized in CdTb_1−*x*_Sm_*x*_GaB_2_O_7_ by controlling Sm^3+^ doping amount and choosing the appropriate excitation wavelength, and this material has potential application prospects as a color-tunable phosphor for w-LEDs.

### 3.9. Quantum yield and thermal stability

Quantum yield (QY) serves as a critical parameter to evaluate the luminescence properties of phosphors, which can be measured based on the following equation:
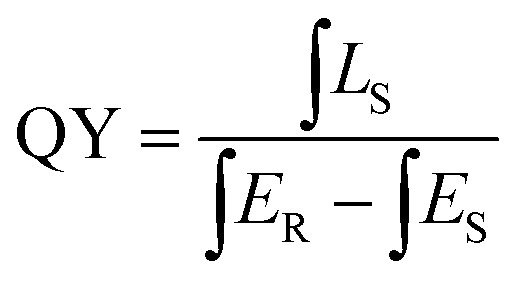
where ∫*L*_S_ is the integrated emission profile of the sample, ∫*E*_R_ and ∫*E*_S_ represent the integrated excitation profile without and with the sample in the integrating sphere, respectively. The excitation and emission spectra of the phosphor CdTb_0.995_Sm_0.005_GaB_2_O_7_ and the reference sample collected in an integrating sphere (*λ*_e*x*_ = 370 nm) are displayed in Fig. S6,† which shows a QY of ∼13.22%. The relatively low QY may be due to the severe aggregation of phosphor particles. We believe that higher QY could be obtained by controlling the particle size, size distribution, morphology and crystalline defects *via* optimization of the preparation conditions or exploration of alternative synthetic routes. This will be investigated in our future work.

**Fig. 9:**
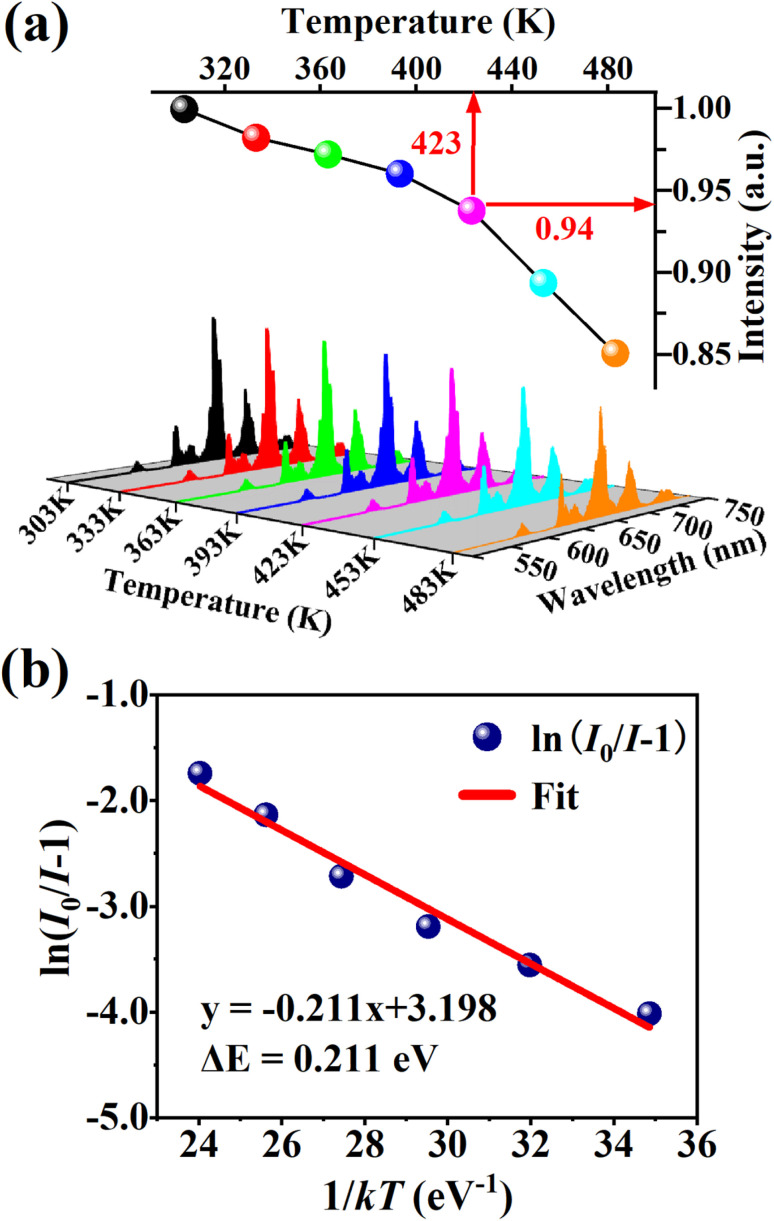
(a) PL spectra and normalized PL intensities of CdTb_0.995_Sm_0.005_GaB_2_O_7_ under different temperatures within 303–483 K (*λ*_ex_ = 370 nm). (b) Relationship of ln(*I*_0_/*I* − 1) *versus* 1/*kT* for this phosphor.

Thermal stability is one of the most important prerequisites for the synthesized phosphor to be used in a LED, as temperature greatly affects the brightness and color output. Fig. 9(a) exhibits the temperature-dependent PL spectra of the CdTb_0.995_Sm_0.005_GaB_2_O_7_ sample upon 370 nm excitation. Obviously, the position and shape of emission peaks remain almost unchanged, while the emission intensity diminishes with increasing temperature due to thermal quenching caused by non-radiative transitions. Furthermore, the emission intensities are integrated over the spectral range of 450–750 nm, and are normalized as compared to the case of 303 K. The results in the inset of Fig. 9(a) show that the relative emission intensity of CdTb_0.995_Sm_0.005_GaB_2_O_7_ at 423 K (the temperature at which LEDs typically operate) remains about 94% of that at 303 K. Compared with some previously reported phosphors, such as Ba_3_BiPbEuO(BO_3_)_4_ (37%), Ba_2_Lu_4.48_Eu_0.5_La_0.02_B_5_O_17_ (38.2%) and LaMgAl_11_O_19_ : 0.05Sm^3+^, 0.2Eu^3+^ (<60%),^59,67,68^ the thermal stability of CdTb_0.995_Sm_0.005_GaB_2_O_7_ seems to be better. For further understanding the influence of temperature on luminescence, the activation energy of thermal quenching (Δ*E*) was measured on the basis of the modified Arrhenius equation^69^:
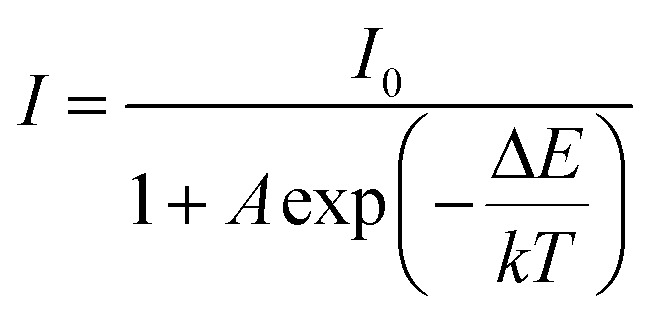
where *I* and *I*_0_ are related to the PL intensity at a given temperature *T* and initial temperature, respectively, *A* is a constant for the host, and *k* stands for the Boltzmann coefficient (8.62 × 10^−5^ eV K^−1^). Upon specific rearrangement, the above equation can be rewritten as:
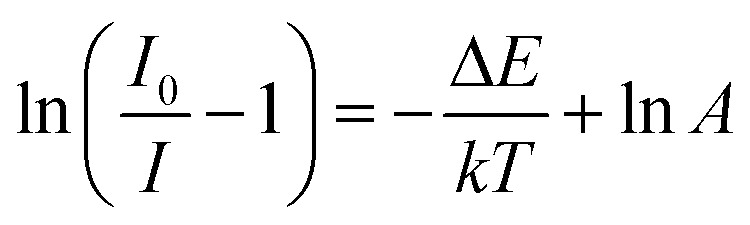


Fig. 9(b) shows the relationship between ln[(*I*_0_/*I*) − 1] and 1/*kT* of the phosphor. It can be seen that the plot can be well fitted to a straight line with the correlation coefficient *R*^2^ = 0.972 and the slope of −0.211, so the activation energy Δ*E* is 0.211 eV. The relatively high activation energy obtained in this work indicates that this phosphor possesses good color thermal stability and can be recommended as a suitable candidate for high-power LED applications.

## 4. Conclusions

CdTbGaB_2_O_7_ is the first quaternary compound found in the CdO–Ln_2_O_3_–Ga_2_O_3_–B_2_O_3_ (Ln = rare-earth metals) system. It has a melilite-type structure, in which [B_2_O_7_]^8−^ tetrahedral dimers and [GaO_4_]^5−^ tetrahedra share corners to generate 2D [Ga(B_2_O_7_)]_*n*_^5*n*−^ layers that are further bridged by 8-coordinated (Cd^2+^/Tb^3+^) cations giving rise to a 3D framework. The IR and Raman studies supported the presence of B_2_O_7_ tetrahedral dimers and GaO_4_ groups. The UV-vis absorption measurement of CdTbGaB_2_O_7_ showed the typical Tb^3+^ absorption peaks and the band gaps of 4.10 and 4.33 eV for indirect and direct transitions, respectively. Furthermore, a series of CdTb_1−*x*_Sm_*x*_GaB_2_O_7_ (0 ≤ *x* ≤ 0.2) phosphors was synthesized at 800 °C. Under the irradiation of 370 nm, the emission lines of Tb^3+^: ^5^D_4_ → ^7^F_6,5,4,3_ and Sm^3+^: ^4^G_5/2_ → ^6^H_5/2,7/2,9/2,11/2_ appeared simultaneously in the PL spectra of CdTb_1−*x*_Sm_*x*_GaB_2_O_7_. The emission intensity of Tb^3+^ at 545 nm decreased monotonously, while that of Sm^3+^ at 603 nm first increased and then declined with increasing Sm^3+^ content, showing the Tb^3+^ → Eu^3+^ energy transfer and the concentration quenching between Sm^3+^ ions. The optimized CdTb_0.995_Sm_0.005_GaB_2_O_7_ phosphor exhibited a QY of 13.22% and good thermal stability with Δ*E* of 0.211 eV. The luminescence color can be tuned from green to orange-red by changing the Sm^3+^ doping concentration, and these types of materials have potential as tunable luminescence materials to meet the application requirements for n-UV LEDs.

## Conflicts of interest

There are no conflicts to declare.

## Supplementary Material

RA-013-D3RA03002D-s001

RA-013-D3RA03002D-s002
