# Supplementary material for: A new melilite-type rare-earth borate CdTbGaB2O7 and multicolor tunable emission in the CdTb1−xSmxGaB2O7 (0 ≤ x ≤ 0.2) phosphors
Source: RSC Adv. 2023 Jun 1;13(24):16272–84. doi: 10.1039/d3ra03002d (PMC10234147; doi:10.1039/d3ra03002d)
Supplement: RA-013-D3RA03002D-s001 [file RA-013-D3RA03002D-s001.pdf]

Electronic Supplementary Material (ESI) for RSC Advances.
This journal is © The Royal Society of Chemistry 2023

Supporting Information

A new melilite-type rare-earth borate $CdTbGaB_2O_7$ and multicolor tunable emission in the $CdTb_{1-x}Sm_xGaB_2O_7$ ($0 \leq x \leq 0.2$) phosphors

Xuean Chen[a,*], Jinyuan Zhang[a], Weiqiang Xiao[b] and Xiaoyan Song [a]

[a]*Faculty of Materials and Manufacturing, Key Laboratory of Advanced Functional Materials, Ministry of Education of China, Beijing University of Technology, 100124 Beijing, China*
[b]*Beijing Key Laboratory of Microstructure and Property of Solids, Beijing University of Technology, 100124 Beijing, China*

Contents:

1. **Table S1** Atomic coordinates, site occupancies, and equivalent isotropic displacement parameters U_{eq} (Å^2) of $CdTbGaB_2O_7$ with standard deviations in parentheses.
2. **Table S2** Anisotropic displacement parameters U_{ij} (Å^2) of $CdTbGaB_2O_7$ with standard deviations in parentheses.
3. **Table S3** Selected bond lengths (Å) and angles (°) for $CdTbGaB_2O_7$.
4. **Table S4** Correlation table of the internal and external modes of B_2O_7 "pyro" units between the site group of the molecule (C_{2v}) and the factor group of the crystal (D_{2d}).
5. **Table S5** Rietveld refinement results of $CdTb_{1-x}Sm_xGaB_2O_7$ ($0 \leq x \leq 1$).
6. **Table S6** Atomic coordinates, occupancy factors, and isotropic thermal displacement factors obtained from the Rietveld refinements for $CdTb_{1-x}Sm_xGaB_2O_7$ ($0 \leq x \leq 1$).
7. **Fig. S1** A FE-SEM image, typical EDX results, and elemental mapping of the $CdTbGaB_2O_7$ single-crystal.
8. **Fig. S2** Infrared and Raman spectra of $CdTbGaB_2O_7$ and $CdSmGaB_2O_7$.
9. **Fig. S3** UV–vis absorption spectra of $CdTbGaB_2O_7$ (a) and $CdSmGaB_2O_7$ (b).
10. **Fig. S4** Rietveld refinements of powder X-ray diffractograms of the $CdTb_{1-x}Sm_xGaB_2O_7$ (x = 0.001, 0.002, 0.005, 0.01, 0.05, 0.1, and 0.2) solid solutions.
11. **Fig. S5** Survey (a) and core-level spectra of Cd 3d (b), Tb 3d (c), Sm 4d (d), Ga 3p (e), B 1s (f) and O 1s (g) for $CdTb_{0.995}Sm_{0.005}GaB_2O_7$.
12. **Fig. S6** The excitation line of $BaSO_4$ reference and the PL spectrum of the $CdTb_{0.995}Sm_{0.005}GaB_2O_7$ phosphor collected using an integrating sphere (λ_{ex} = 370 nm).

Table S1

Atomic coordinates, site occupancies, and equivalent isotropic displacement parameters U_{eq} ($Å^2$) of $CdTbGaB_2O_7$ with standard deviations in parentheses.

Atoms	Wyck sites	Site symmetry	X	Y	Z	Occupancies	U_{eq}
Cd1/Tb1	4e	C_s	0.83827(3)	0.33827(3)	0.49097(7)	0.5/0.5	0.00997(10)
Ga1	2a	S_4	1.0000	0.0000	0.0000	1	0.00576(14)
B1	4e	C_s	0.8658(6)	0.6343(6)	0.9369(12)	1	0.0069(9)
O1	2c	C_{2v}	1.0000	0.5000	0.8248(12)	1	0.0119(12)
O2	8f	C_1	0.8095(4)	0.0798(4)	0.2140(7)	1	0.0109(6)
O3	4e	C_s	0.8568(5)	0.6432(5)	0.2366(8)	1	0.0139(8)

Note: U_{eq} is defined as one third of the trace of the orthogonalized U_{ij} tensor.

Table S2

Anisotropic displacement parameters U_{ij} ($Å^2$) of $CdTbGaB_2O_7$ with standard deviations in parentheses.

Atoms	U_{11}	U_{22}	U_{33}	U_{23}	U_{13}	U_{12}
Cd1/Tb1	0.01079(11)	0.01079(11)	0.00832(13)	-0.00376(10)	-0.00376(10)	0.00176(10)
Ga1	0.00614(19)	0.00614(19)	0.0050(3)	0.000	0.000	0.000
B1	0.0068(13)	0.0068(13)	0.007(2)	0.0001(12)	-0.0001(12)	-0.0015(17)
O1	0.0154(18)	0.0154(18)	0.005(2)	0.000	0.000	0.013(3)
O2	0.0088(13)	0.0155(14)	0.0085(11)	-0.0030(10)	0.0009(11)	0.0032(11)
O3	0.0190(14)	0.0190(14)	0.0036(14)	-0.0020(10)	0.0020(10)	0.0059(19)

Table S3

Selected bond lengths (Å) and angles (°) for $CdTbGaB_2O_7$.

Cd1/Tb1-O1	2.305(4)	Ga1-O2 × 4	1.824(3)
Cd1/Tb1-O2 × 2	2.316(3)		
Cd1/Tb1-O3	2.401(4)	B1-O3	1.419(7)
Cd1/Tb1-O2 × 2	2.505(3)	B1-O1	1.492(7)
Cd1/Tb1-O3 × 2	2.547(3)	B1-O2 × 2	1.525(5)
Mean	2.430	Mean	1.490
O2-Ga1-O2 × 4	107.90(10)	O3-B1-O2 × 2	116.1(3)
O2-Ga1-O2 × 2	112.7(2)	O1-B1-O2 × 2	102.6(3)
Mean	109.5	O2-B1-O2	102.9(4)
O3-B1-O1	114.6(5)	Mean	109.15

Table S4

Correlation table of the internal and external modes of B_2O_7 "pyro" units between the site group of the molecule (C_{2v}) and the factor group of the crystal (D_{2d}).

Species	Assignment	site group symmetry	Factor group symmetry
		C_{2v}	D_{2d}
	$\nu_s(BO_3)$	A_1	$A_1 + B_2$
		B_1	E
	$\nu_{as}(BO_3)$	A_1	$A_1 + B_2$
		A_2	$A_2 + B_1$
		B_1	E
		B_2	E
B_2O_7	$\nu_s(B^OB)$	A_1	$A_1 + B_2$
	$\nu_{as}(B^OB)$	B_1	E
	$\delta(B^OB)$	A_1	$A_1 + B_2$
	$\rho(BO_3)$	A_2	$A_2 + B_1$
		B_2	E
	$\delta(BO_3)$	$3A_1 + 2A_2 + 3B_1 + 2B_2$	$3A_1 + 2A_2 + 2B_1 + 3B_2 + 5E$
	$T'(B_2O_7)$	$A_1 + B_1 + B_2$	$A_1 + B_2 + 2E$
	$L(B_2O_7)$	$A_2 + B_1 + B_2$	$A_2 + B_1 + 2E$
	**Γ**	$\mathbf{8A_1 + 5A_2 + 8B_1 + 6B_2}$	$\mathbf{8A_1 + 5A_2 + 5B_1 + 8B_2 + 14E}$

Note: ν_s, symmetric stretching; ν_{as}, antisymmetric stretching; δ, bending; ρ, rocking; T′, translational mode; L, librational mode; Γ, irreducible representation.

Table S5

Rietveld refinement results of $CdTb_{1-x}Sm_xGaB_2O_7$ ($0 \leq x \leq 1$).

Sm^{3+} content	x=0	x=0.001	x=0.002	x=0.005	x=0.01	x=0.05	x =0.1	x =0.2	x =1
Space group	P**Error!**2_1m	P**Error!**2_1m	P**Error!**2_1m	P**Error!**2_1m	P**Error!**2_1m	P**Error!**2_1m	P**Error!**2_1m	P**Error!**2_1m	P**Error!**2_1m
a (Å)	7.34374(10)	7.34419(13)	7.34398(9)	7.34405(8)	7.34421(12)	7.34551(13)	7.34683(12)	7.34907(12)	7.37531(10)
c (Å)	4.72012(8)	4.72007(10)	4.72025(7)	4.72030(7)	4.72100(10)	4.72210(10)	4.72337(10)	4.72619(10)	4.75700(8)
V (Å^3)	254.558(8)	254.587(10)	254.582(7)	254.590(7)	254.639(10)	254.788(11)	254.948(10)	255.257(10)	258.758(9)
2θ range (°)	5-80	5-80	5-80	5-80	5-80	5-80	5-80	5-80	5-80
R_p, %	2.30	2.54	2.34	2.33	2.63	2.68	2.54	2.66	3.02
R_{wp}, %	3.51	3.91	3.56	3.51	4.09	4.23	3.92	4.00	4.91
GOF	2.03	2.14	2.00	2.02	2.40	2.43	2.21	2.33	2.45

Table S6

Atomic coordinates, occupancy factors, and isotropic thermal displacement factors obtained from the Rietveld refinements for $CdTb_{1-x}Sm_xGaB_2O_7$ ($0 \le x \le 1$).

$CdTbGaB_2O_7$	*x*	*y*	*z*	Occ.	B_{eq}
Cd1/Tb1	0.83725(14)	0.33725(14)	0.4890(7)	0.5/0.5	0.7872
Ga1	1.00000	0.00000	0.00000	1	0.4548
B1	0.86580	0.63430	0.93690	1	0.5448
O1	1.00000	0.50000	0.82480	1	0.9396
O2	0.80950	0.07980	0.21400	1	0.8606
O3	0.85680	0.64320	0.23660	1	1.098
$CdTb_{0.999}Sm_{0.001}GaB_2O_7$	*x*	*y*	*z*	Occ.	B_{eq}
Cd1/Tb1/Sm1	0.83715(18)	0.33715(18)	0.4883(9)	0.5/0.4995/0.0005	0.7872
Ga1	1.00000	0.00000	0.00000	1	0.4548
B1	0.86580	0.63430	0.93690	1	0.5448
O1	1.00000	0.50000	0.82480	1	0.9396
O2	0.80950	0.07980	0.21400	1	0.8606
O3	0.85680	0.64320	0.23660	1	1.098
$CdTb_{0.998}Sm_{0.002}GaB_2O_7$	*x*	*y*	*z*	Occ.	B_{eq}
Cd1/Tb1/Sm1	0.83727(14)	0.33727(14)	0.4888(7)	0.5/0.499/0.001	0.7872
Ga1	1.00000	0.00000	0.00000	1	0.4548
B1	0.86580	0.63430	0.93690	1	0.5448
O1	1.00000	0.50000	0.82480	1	0.9396
O2	0.80950	0.07980	0.21400	1	0.8606
O3	0.85680	0.64320	0.23660	1	1.098
$CdTb_{0.995}Sm_{0.005}GaB_2O_7$	*x*	*y*	*z*	Occ.	$U_{iso.}$
Cd1/Tb1/Sm1	0.83748(16)	0.33748(16)	0.4909(7)	0.5/0.4975/0.0025	0.7872
Ga1	1.00000	0.00000	0.00000	1	0.4548
B1	0.86580	0.63430	0.93690	1	0.5448
O1	1.00000	0.50000	0.82480	1	0.9396
O2	0.80950	0.07980	0.21400	1	0.8606
O3	0.85680	0.64320	0.23660	1	1.098
$CdTb_{0.99}Sm_{0.01}GaB_2O_7$	*x*	*y*	*z*	Occ.	$U_{iso.}$
Cd1/Tb1/Sm1	0.83705(17)	0.33705(17)	0.4877(8)	0.5/0.495/0.005	0.7872
Ga1	1.00000	0.00000	0.00000	1	0.4548
B1	0.86580	0.63430	0.93690	1	0.5448
O1	1.00000	0.50000	0.82480	1	0.9396
O2	0.80950	0.07980	0.21400	1	0.8606
O3	0.85680	0.64320	0.23660	1	1.098

$CdTb_{0.95}Sm_{0.05}GaB_2O_7$	*x*	*y*	*z*	Occ.	$U_{iso.}$
Cd1/Tb1/Sm1	0.83707(18)	0.33707(18)	0.4885(9)	0.5/0.475/0.025	0.7872
Ga1	1.00000	0.00000	0.00000	1	0.4548
B1	0.86580	0.63430	0.93690	1	0.5448
O1	1.00000	0.50000	0.82480	1	0.9396
O2	0.80950	0.07980	0.21400	1	0.8606
O3	0.85680	0.64320	0.23660	1	1.098
$CdTb_{0.9}Sm_{0.1}GaB_2O_7$	*x*	*y*	*z*	Occ.	$U_{iso.}$
Cd1/Tb1/Sm1	0.83705(17)	0.33705(17)	0.4873(8)	0.5/0.45/0.05	0.7872
Ga1	1.00000	0.00000	0.00000	1	0.4548
B1	0.86580	0.63430	0.93690	1	0.5448
O1	1.00000	0.50000	0.82480	1	0.9396
O2	0.80950	0.07980	0.21400	1	0.8606
O3	0.85680	0.64320	0.23660	1	1.098
$CdTb_{0.8}Sm_{0.2}GaB_2O_7$	*x*	*y*	*z*	Occ.	$U_{iso.}$
Cd1/Tb1/Sm1	0.83706(17)	0.33706(17)	0.4888(8)	0.5/0.4/0.1	0.7872
Ga1	1.00000	0.00000	0.00000	1	0.4548
B1	0.86580	0.63430	0.93690	1	0.5448
O1	1.00000	0.50000	0.82480	1	0.9396
O2	0.80950	0.07980	0.21400	1	0.8606
O3	0.85680	0.64320	0.23660	1	1.098
$CdSmGaB_2O_7$	*x*	*y*	*z*	Occ.	$U_{iso.}$
Cd1/Sm1	0.83681(18)	0.33681(18)	0.4892(9)	0.5/0.5	0.7872
Ga1	1.00000	0.00000	0.00000	1	0.4548
B1	0.86580	0.63430	0.93690	1	0.5448
O1	1.00000	0.50000	0.82480	1	0.9396
O2	0.80950	0.07980	0.21400	1	0.8606
O3	0.85680	0.64320	0.23660	1	1.098

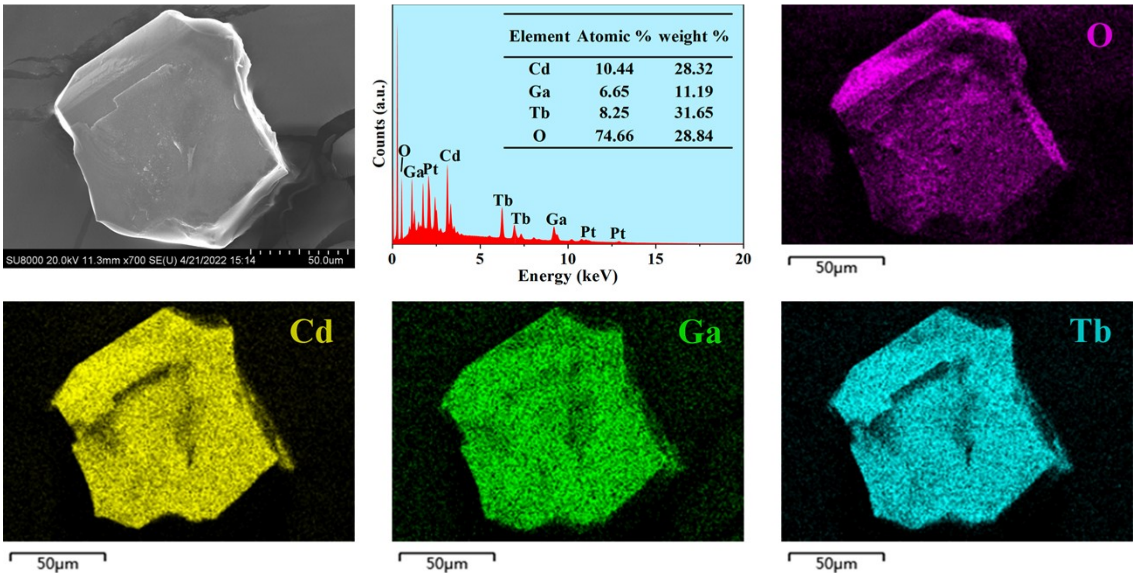

Fig. S1 A FE-SEM image, typical EDX results, and elemental mapping of the $CdTbGaB_2O_7$ single-crystal. Pt element comes from the pretreatment process (A thin layer of Pt was evaporated on the sample surface to provide electrical conductivity).

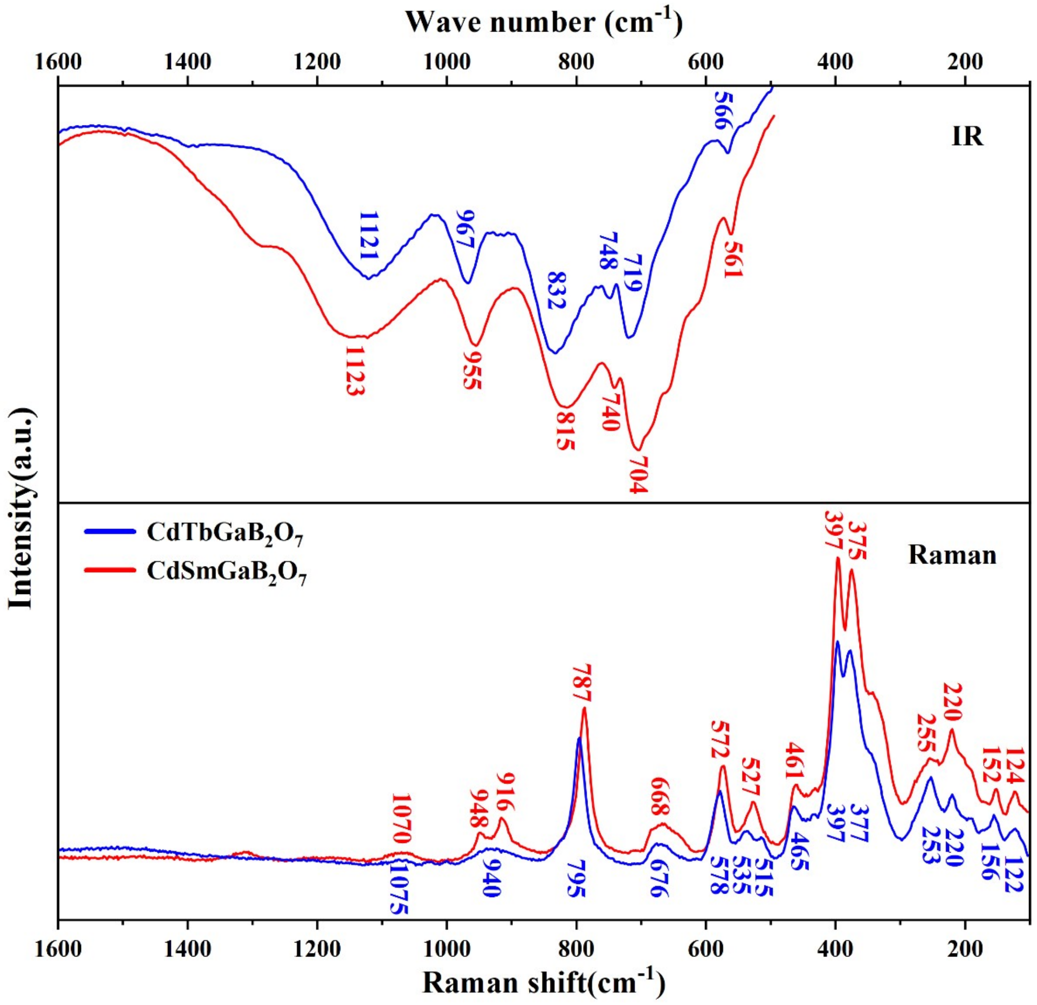

Fig. S2 Infrared and Raman spectra of $CdTbGaB_2O_7$ and $CdSmGaB_2O_7$.

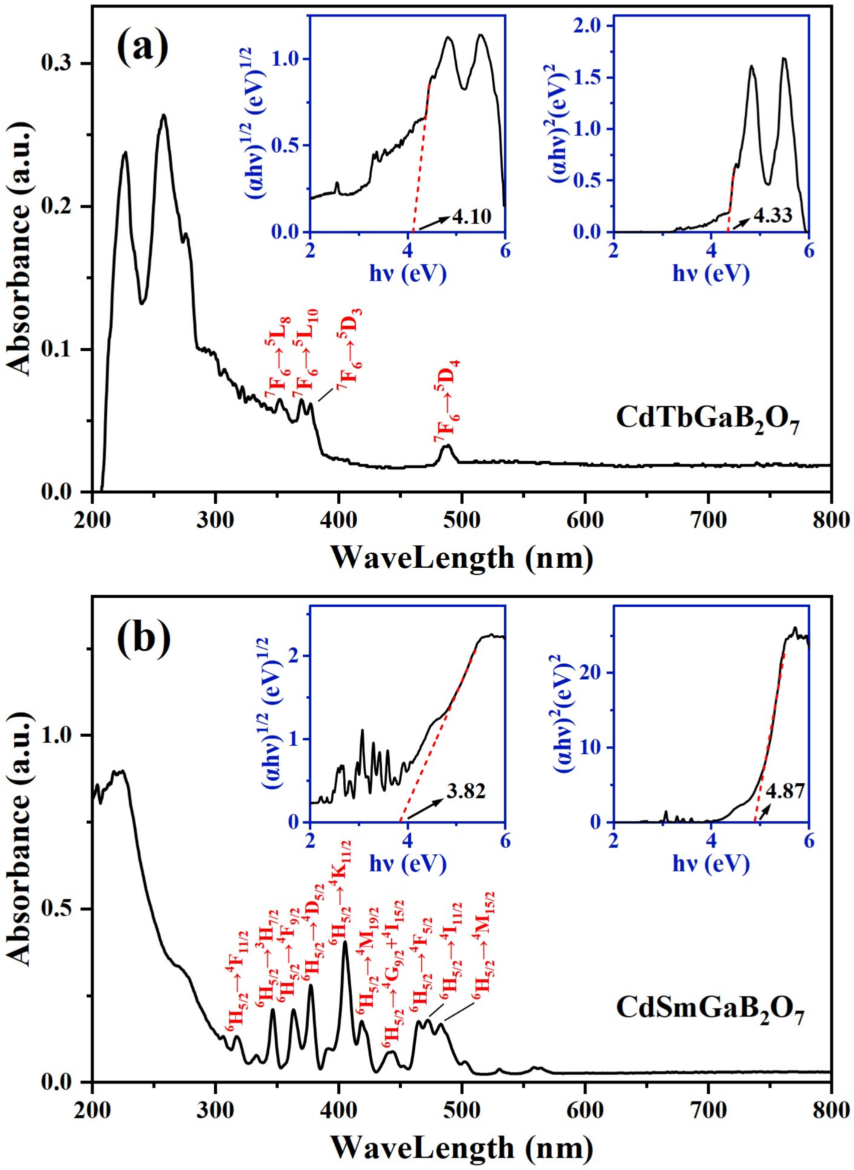

Fig. S3 UV–vis absorption spectra of $CdTbGaB_2O_7$ (a) and $CdSmGaB_2O_7$ (b). The insets show the Tauc plots from indirect (left) and direct (right) transitions.

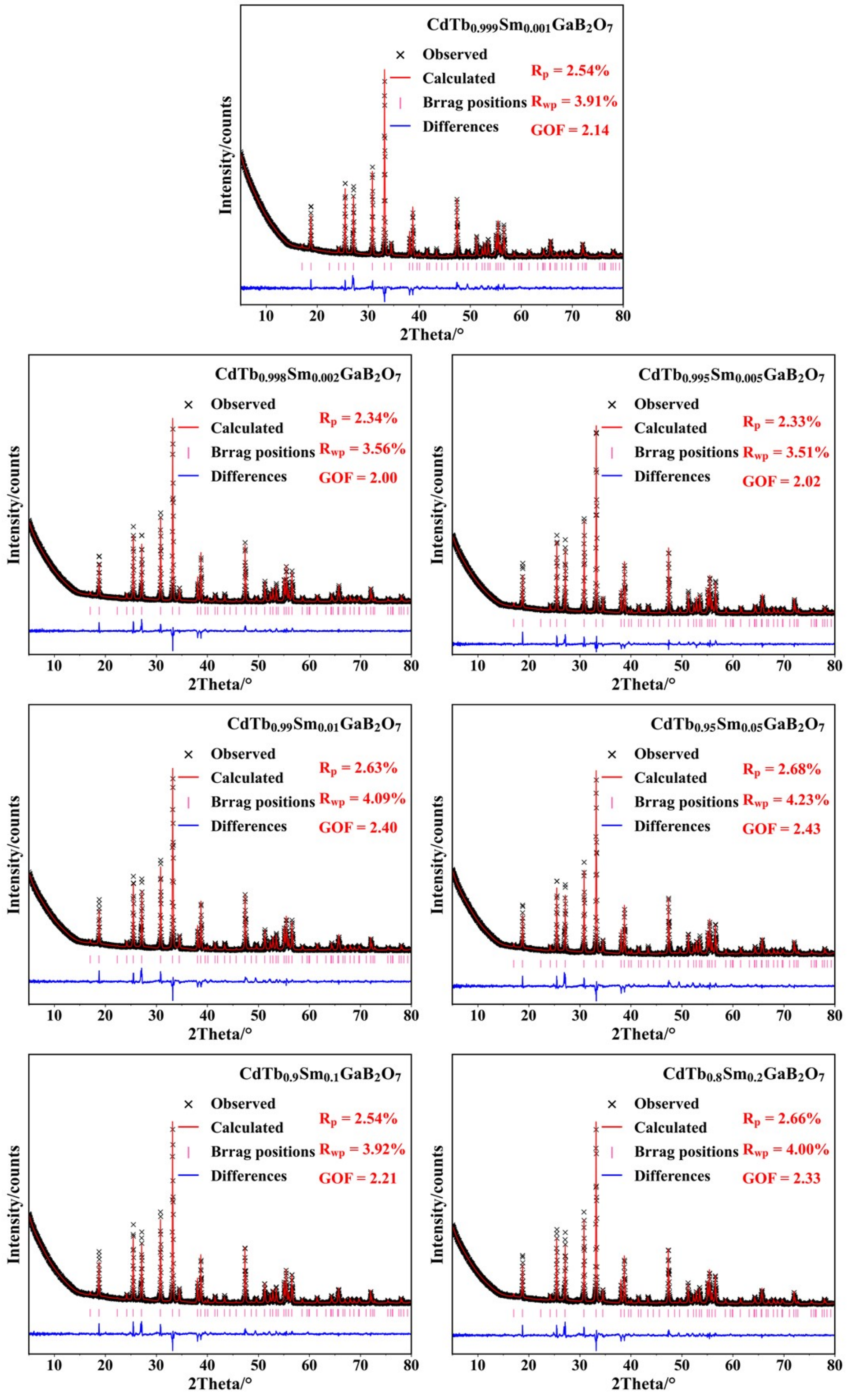

Fig. S4 Rietveld refinements of powder X-ray diffractograms of the $CdTb_{1-x}Sm_xGaB_2O_7$ (x = 0.001, 0.002, 0.005, 0.01, 0.05, 0.1, and 0.2) solid solutions.

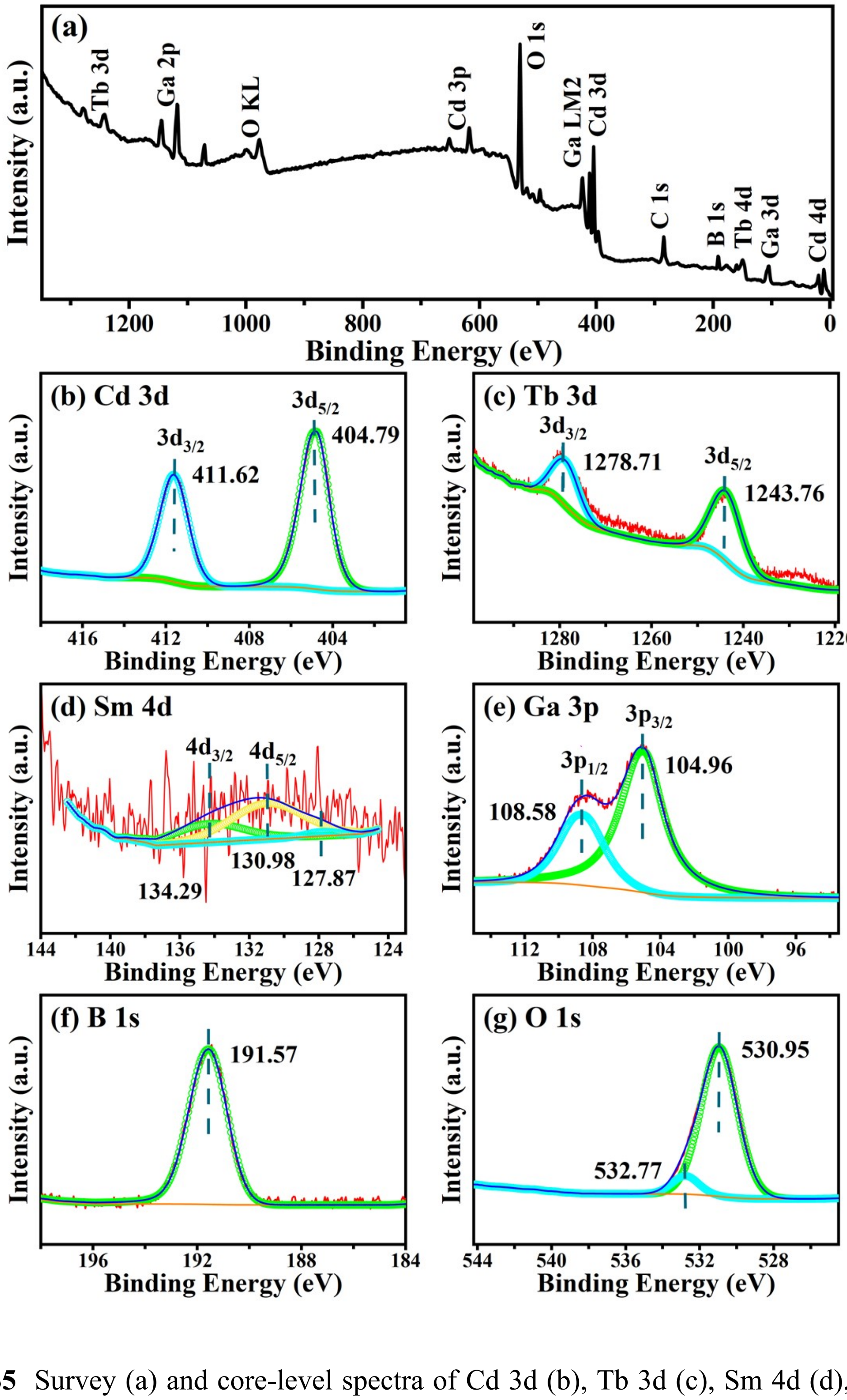

Fig. S5 Survey (a) and core-level spectra of Cd 3d (b), Tb 3d (c), Sm 4d (d), Ga 3p (e), B 1s (f) and O 1s (g) for $CdTb_{0.995}Sm_{0.005}GaB_2O_7$.

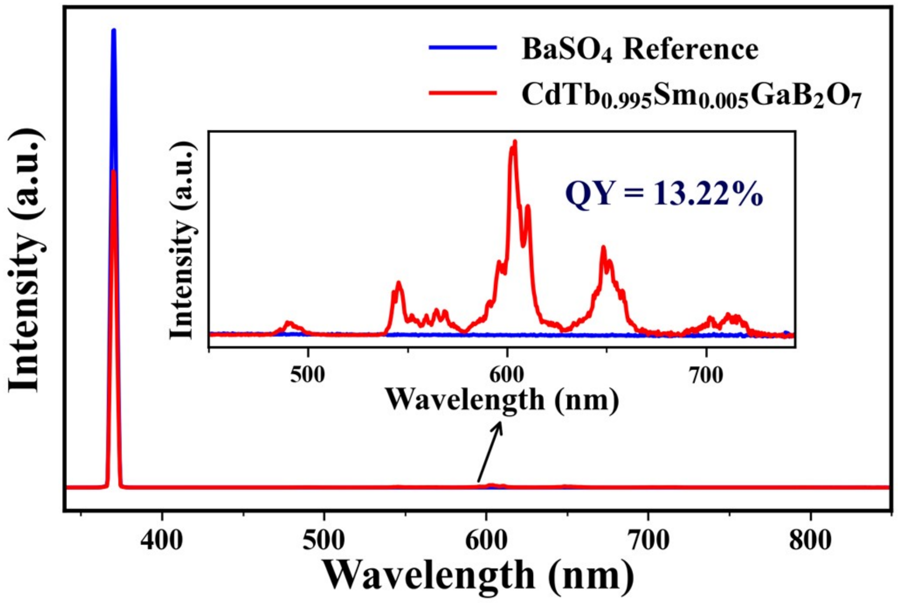

Fig. S6 The excitation line of $BaSO_4$ reference and the PL spectrum of the $CdTb_{0.995}Sm_{0.005}GaB_2O_7$ phosphor collected using an integrating sphere (λ_{ex} = 370 nm); inset shows a local magnification of the PL spectrum.
